# Alk1 and Alk5 inhibition by Nrp1 controls vascular sprouting downstream of Notch

**DOI:** 10.1038/ncomms8264

**Published:** 2015-06-17

**Authors:** Irene Maria Aspalter, Emma Gordon, Alexandre Dubrac, Anan Ragab, Jarek Narloch, Pedro Vizán, Ilse Geudens, Russell Thomas Collins, Claudio Areias Franco, Cristina Luna Abrahams, Gavin Thurston, Marcus Fruttiger, Ian Rosewell, Anne Eichmann, Holger Gerhardt

**Affiliations:** 1 Vascular Biology Laboratory, London Research Institute, Cancer Research UK, London WC2A 3LY, UK; 2 Yale Cardiovascular Research Center, Yale University School of Medicine, New Haven, Connecticut 06510, USA; 3 Transgenic Services, London Research Institute—Clare Hall Laboratories, Cancer Research UK, Potters Bar EN6 3LD, UK; 4 Developmental Signalling Laboratory, London Research Institute, Cancer Research UK, London WC2A 3LY, UK; 5 Epigenetic Events in Cancer, Centre for Genomic Regulation and Universitat Pompeu Fabra, Barcelona 080003, Spain; 6 Vascular Patterning Laboratory, Vesalius Research Center, VIB, KU Leuven B-3000, Belgium; 7 Max-Delbrück-Center for Molecular Medicine, Robert-Rössle-Strasse 10, Berlin 13125, Germany; 8 Regeneron Pharmaceuticals, Tarrytown, New York 10591, USA; 9 UCL Institute of Ophthalmology, University College London, London EC1V 9EL, UK; 10 CIRB Collège de France, Inserm U1050, Paris 75231, France; 11 Department of Cellular and Molecular Physiology, Yale University Medical School, New Haven, Connecticut 06520, USA

## Abstract

Sprouting angiogenesis drives blood vessel growth in healthy and diseased tissues. Vegf and Dll4/Notch signalling cooperate in a negative feedback loop that specifies endothelial tip and stalk cells to ensure adequate vessel branching and function. Current concepts posit that endothelial cells default to the tip-cell phenotype when Notch is inactive. Here we identify instead that the stalk-cell phenotype needs to be actively repressed to allow tip-cell formation. We show this is a key endothelial function of neuropilin-1 (Nrp1), which suppresses the stalk-cell phenotype by limiting Smad2/3 activation through Alk1 and Alk5. Notch downregulates Nrp1, thus relieving the inhibition of Alk1 and Alk5, thereby driving stalk-cell behaviour. Conceptually, our work shows that the heterogeneity between neighbouring endothelial cells established by the lateral feedback loop of Dll4/Notch utilizes Nrp1 levels as the pivot, which in turn establishes differential responsiveness to TGF-β/BMP signalling.

## Abstract (web summary)

Notch signals are crucial for organization of angiogenic sprouting cells into the leading ‘tip' and trailing ‘stalk' cells. Here the authors show that endothelial neuropilin-1 quantitatively inhibits TGF-β/BMP signalling, explaining how Notch-mediated regulation of neuropilin-1 specifies endothelial tip and stalk cells.

## Graphical abstract

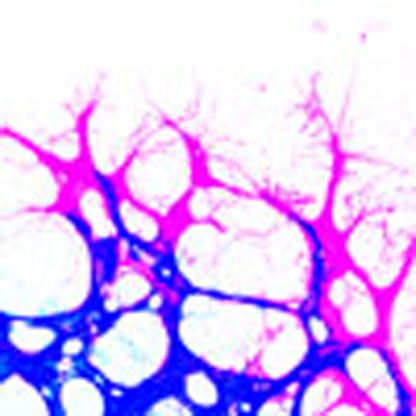

Vascular sprouting is critical for the formation of blood vessel networks during embryonic development and in the physiological response to hypoxia or tissue injury in the adult^1^. Activated endothelial cells display heterogeneous gene expression, morphology and cell behaviour as they collectively engage in the morphogenetic process of sprout formation^2^. Vascular endothelial growth factor A (Vegf-A) stimulates endothelial migration and proliferation^3^, but while some form polarized filopodia protrusions and acquire the leading tip position in the nascent sprout (tip cells), neighbouring cells contribute to elongation and stability of the new vessel (stalk cells)^4^. The specification of tip and stalk cells is regulated by Dll4/Notch signalling^1^. Genetic and pharmacological studies led to the current concept that the tip cell phenotype is the default response of endothelial cells, whereas the stalk cell is actively inhibited from forming tip cells through the activity of Notch. Mosaic analysis illustrated that cells deficient in Notch signalling dominate the tip position, whereas Notch-activated cells are excluded from the tip position^5^,^6^. Dynamic observations identified that endothelial cells compete for the tip cell position by virtue of differential Vegfr levels under control of Dll4/Notch signalling^7^,^8^. All three Vegf receptors contribute to tip/stalk cell competition, but Notch signalling inhibition normalizes the contribution of Vegfr-deficient cells at the tip, indicating that Vegf receptors act upstream of Notch.

Notch downstream effectors driving stalk cell behaviour are attractive targets to prevent excessive angiogenesis in cancer and ocular neovascular disease. A recent study has suggested that the Sox17 transcription factor promotes stalk cell behaviour downstream of Notch; however, another study using similar genetic mouse models reports opposite findings, indicating that the role of Sox17 in tip–stalk cell specification and its relationship with Notch1 may be context dependent^9^.

In parallel to Notch, Bmp/Smad1/5 signalling prominently affects tip/stalk specification. Inhibition of Alk1 or endothelial-specific Smad1 and Smad5 deletion leads to hypersprouting in embryonic and postnatal development. Bmp9 and Bmp-10 inhibit sprouting angiogenesis by activating Notch downstream target genes leading to stalk cell specification^10^,^11^,^12^,^13^. Tgf-β also inhibits sprouting angiogenesis but exhibits differential effects depending on the involvement of the Tgf-β type-1 receptors Alk1 or Alk5 (refs 14, 15, 16, 17, 18). The identity of the signal required to turn Bmp/Alk1 signalling off in tip cells, and whether Alk5 signalling regulates tip/stalk specification, remains unknown. Furthermore, the link between Notch and the Smad signalling pathway in tip/stalk competition has not been identified.

Neuropilin-1 (Nrp1) is a transmembrane receptor that binds several structurally and functionally unrelated classes of ligands, including class-3 semaphorins, Vegf family members and Tgf-β^19^,^20^. Identified as a co-receptor for semaphorin/PlexinD signalling^21^ and Vegf-A/Vegfr2 signalling^22^, it is expressed in the angiogenic vasculature including the tip cells and has been implicated in tip cell function and guidance in the embryo^23^,^24^,^25^,^26^. Nrp1 can enhance Vegf-driven signalling through Vegfr2 via interactions between synectin, an adaptor that binds the PDZ-binding site in the Nrp1 cytoplasmic domain, and Vegfr2 (ref. 27). Following Vegf binding, the Nrp1–Synectin–Vegfr2 complex is internalized into endosomal compartments, where Vegfr2 is protected from dephosphorylation, leading to sustained Erk activation and arteriogenesis^28^. Consequently, mice carrying a targeted deletion of the Nrp1 cytoplasmic domain show impaired arteriogenesis^28^. Notably, however, neither Nrp1cyto mice, nor mice deficient for Synectin show any angiogenesis defects^29^,^30^, suggesting that Nrp1 drives sprouting angiogenesis through other signalling mechanisms.

Here we report that Nrp1 functions as a Notch effector and links Notch with Tgf-β/Alk5 and Bmp9/Alk1 signalling to regulate endothelial competition for the tip position. Nrp1 cell autonomously and quantitatively determines the ability of cells to become tip cells even in the absence of Notch signalling. Loss of Nrp1 renders the vasculature completely refractory to the loss of Notch, demonstrating that Nrp1 is the most critical determinant of tip cell formation and function known to date. Loss-of-function and gain-of-function experiments identify that Nrp1 negatively regulates Smad2/3 activation downstream of Tgf-β and Bmp9 signalling. Genetic inactivation of Alk5 and Alk1 rescues the ability of Nrp1-deficient cells to contribute to the tip position. We propose that Notch-mediated differential expression of Nrp1 critically regulates tip/stalk specification and thus vascular branching by modulating the activity of Tgf-β/Alk5 and Bmp9/Alk1 signalling between neighbouring endothelial cells.

## Results

### Differential Nrp1 expression drives tip cell competition

**Figure 1:**
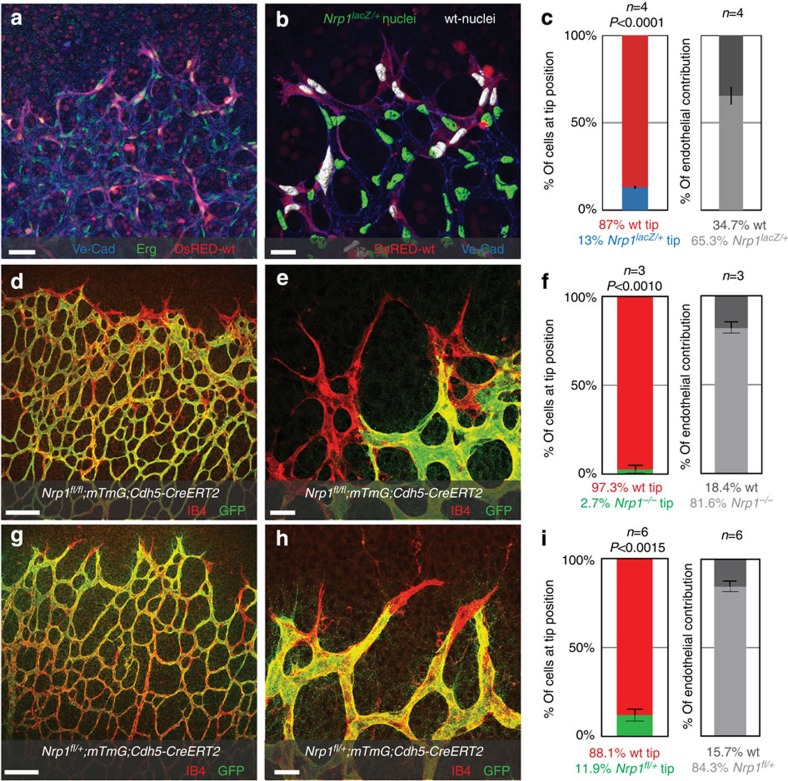
Differential Nrp1 levels affect tip cell competition. (**a**–**c**) Retinal vessels from a wild-type host expressing DsRED injected with *Nrp1*^*Lacz/+*^ ES cells, assayed at postnatal day P5. (**a**) Representative overview of the sprouting front; scale bar, 420 μm. (**b**) Segmented images showing wt nuclei (white) and nuclei from *Nrp1*^*lacZ/+*^ cells (green), using Erg staining; scale bar, 20 μm. (**c**) Quantification of tip cell contribution normalized to overall contribution of cells to the endothelium, *P*<0.0001 compared with wt cells injected retinas. (**d**–**i**) Retinas of *Nrp1*^*fl/fl*^*; mTmG; Cdh5-CreERT2* and *Nrp1*^*fl/+*^*; mTmG; Cdh5-CreERT2* mice injected with 30 μg tamoxifen at P1, retinas were assayed P5. (**d**,**g**) Representative overview of the sprouting front, scale bar, 100 μm, and higher magnification, scale bar, 20 μm (**e**,**h**). Unrecombined, wt cells are labelled with Isolectin-B4 only; recombined Nrp1-deficient cells express GFP. (**f**,**i**) Quantification of recombined Nrp1-deficient cells at the tip, normalized to overall contribution of cells to the endothelium. Statistical significance was determined by comparing the proportion of Nrp1-deficient (green) cells at the tip with the total proportion of Nrp1-deficient cells; *P*<0.001 (**f**), *P*<0.0015 (**i**). *n*=the number of retinas analysed; *n*=4 (**c**), *n*=3 (**f**) and *n*=6 (**i**). Values represent mean±s.e.m. Statistical significance was assessed using a Student's unpaired *t*-test.

To determine the functional importance of Nrp1 expression levels in sprouting angiogenesis and tip cell formation, we established chimeric mice, and chimeric embryoid body (EB) sprouting assays combining cells homozygous or heterozygous for a Nrp1-null/lacZ reporter allele (*Nrp1*^*lacZ/lacZ*^ or *Nrp1*^*lacZ/+*^, respectively) with wt cells, expressing the marker DsRed. *Nrp1*^*lacZ/lacZ*^ or *Nrp1*^*lacZ/+*^ cells were markedly outcompeted by wild-type (wt) cells at the tip position, demonstrating that Nrp1 levels are critical for tip formation (Fig. 1a–c; Supplementary Fig. 1a–c). We also induced endothelial Nrp1 deletion during postnatal retinal angiogenesis by crossing *Nrp1*^*fl/fl*^^31^ and *Cdh5-CreERT2* mice^32^,^33^. Tamoxifen-mediated recombination efficiently reduced Nrp1 protein levels, decreased radial expansion and reduced sprouting/branching (Supplementary Fig. 1d–m), illustrating that Nrp1 is critical for sprouting and branching during postnatal angiogenesis.

Low-dose tamoxifen injection in conditional *Nrp1*^*fl/fl*^*; Cdh5-CreERT2* crossed with mTmG Cre-reporter mice^34^ produces recombination of the *Nrp1* floxed allele and the mTmG reporter allele in a subset of green fluorescent protein (GFP)-positive cells. In samples where around 80% of retinal endothelial cells were GFP positive, and therefore have experienced Cre activity, only 3% of *Nrp1*^*fl/fl*^ homozygous cells are found at the capillary tip (Fig. 1d–f). Moreover, heterozygous *Nrp1*^*fl/+*^*; mTmG; Cdh5-CreERT2* retinas also showed an under-representation of Nrp1 heterozygous cells in the tip position (Fig. 1g–i), despite only a slight decrease in overall vessel branching (Supplementary Fig. 2a–d) and no effect on proliferation (Supplementary Fig. 2e–i).

### Nrp1 drives tip cell competition independent of Dll4

**Figure 2:**
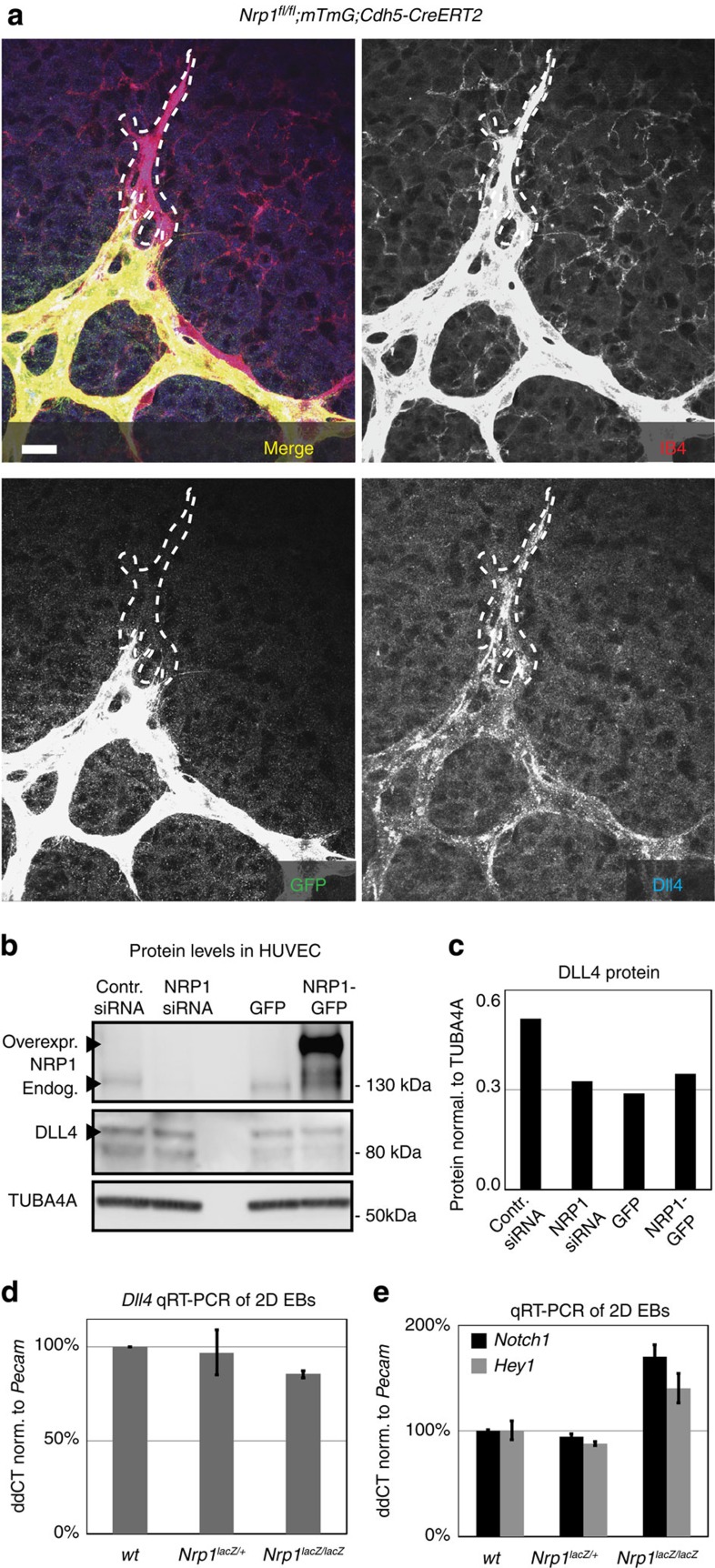
Nrp1-deficient cells express Dll4. (**a**) Representative image of Dll4 expression in endothelial cells at the vascular front of mice carrying endothelial-specific *Nrp1* deletion, scale bar, 20 μm. Mice were injected with 30 μg of tamoxifen at P1. Tip cell outline is indicated by a dashed line. Dll4 expression was comparable between *Nrp1*-deficient, GFP-positive cells and *Nrp1*-positive, GFP-negative cells. (**b**) P4 HUVEC cells transfected with control siRNA, NRP1 siRNA, control-GFP construct and NRP1–GFP–His-construct. NRP1 and DLL4 protein levels were assessed 24 h after transfection by western blot (two individual experiments). Full western blots are shown in Supplementary Fig. 9. (**c**) Quantification of DLL4 protein expression normalized to TUBA4A, a representative of two experiments is shown. (**d**,**e**) Real-time quantitative PCR for *Dll4* (**d**) and *Notch/Hey1* (**e**) from 2D EBs derived from wt cells, *Nrp1*^*lacZ/+*^ cells and *Nrp1*^*lacZ/lacZ*^ cells. Experimental triplicates were performed; represented values were normalized to Pecam expression and wt transcription levels. Values indicate mean±s.d.

As Vegfr2 levels regulate Dll4 (ref. 7), and Nrp1 is considered a co-receptor for Vegf-A/Vegfr-2 (ref. 22), we investigated whether Nrp1 levels influence Dll4 levels. We found no differences in Dll4 staining between Nrp1-expressing and -deficient cells in chimeric EBs, as well as in complete or mosaic deficient retinas (Fig. 2a; Supplementary Fig. 3). Furthermore, overall Dll4 mesenger RNA and protein levels were not significantly affected in *Nrp1*^*lacZ/lacZ*^ EBs and human umbilical vein endothelial cells (HUVECs) transduced with Nrp1 short interfering RNA (siRNA) or overexpressing Nrp1–GFP (Fig. 2b–d), and Notch1 expression and its target Hey1 were unchanged (Fig. 2e). In contrast, inhibiting Notch signalling in HUVECs using the γ-secretase inhibitor DAPT led to upregulation of Nrp1 in a time-dependent manner (Supplementary Fig. 4a,b). Similarly, we observed increased Nrp1 expression in EBs upon DAPT treatment (Supplementary Fig. 4c,d). The Notch-mediated downregulation of Nrp1 together with the observation that already a 50% gene dose reduction renders endothelial cells unable to form tip cells although Dll4 expression is unaffected, suggests that Nrp1 functions not upstream, but as a downstream effector of Notch activity in tip/stalk formation.

### Nrp1 acts as a downstream effector of Notch

**Figure 3:**
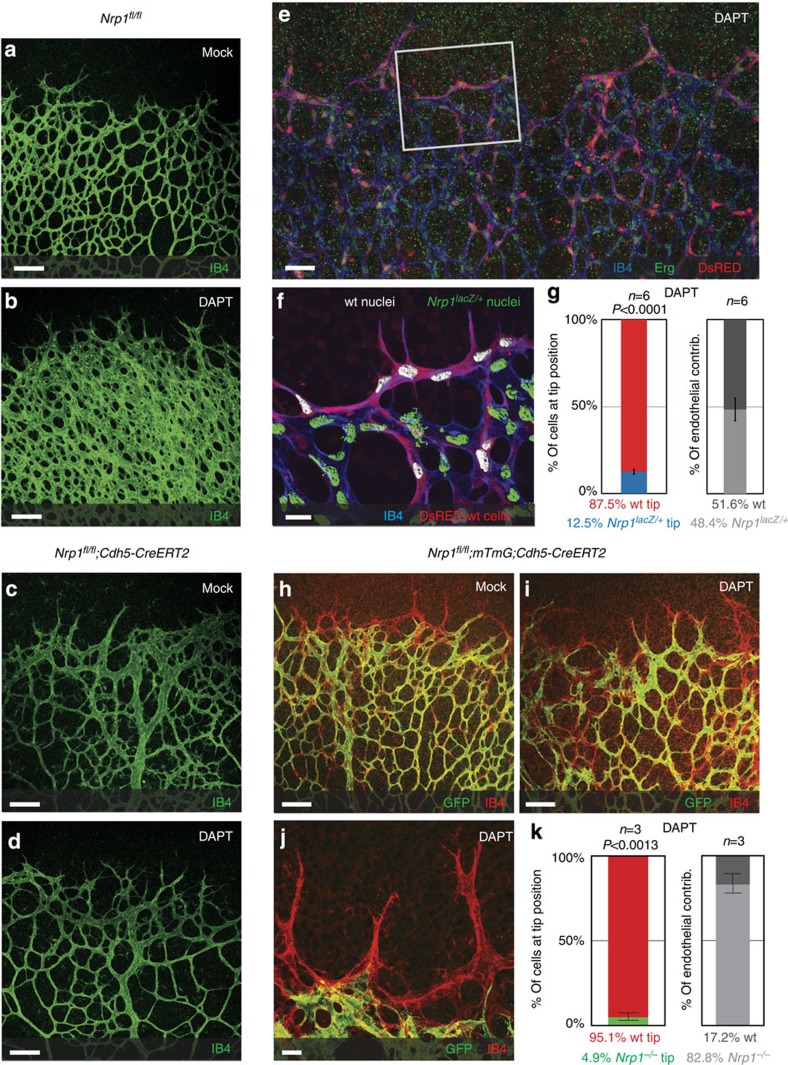
Nrp1 acts as downstream effector of Notch. (**a**–**d**) Representative images of the Isolectin-B4-stained retinal vasculature, of P5 *Nrp1*^*fl/fl*^ and *Nrp1*^*fl/fl*^*; Cdh5-CreERT2* mice, injected with 100 μg tamoxifen at P1/P2 and treated with 100 mg kg^−1^ DAPT at P4; scale bar, 100 μm. (**a**) Retinal vessels of *Nrp1*^*fl/fl*^ mice; (**b**) DAPT treatment of *Nrp1*^*fl/fl*^; (**c**) *Nrp1*^*fl/fl*^*; Cdh5-CreERT2*; (**d**) DAPT-treated *Nrp1*^*fl/fl*^*; Cdh5-CreERT2* mice. *n*=number of retinas examined; *n*=4 (**a**), *n*=4 (**b**), *n*=6 (**c**) and *n*=6 (**d**). (**e**–**g**) P5 retinal vasculature of wt blastocysts expressing DsRED injected with *Nrp1*^*lacZ/+*^ ES cells, treated with DAPT (100 mg kg^−1^) at P4. (**e**) Representative overview of the sprouting front; scale bar, 420 μm. (**f**) Segmented images; wt nuclei (white) and *Nrp1*^*lacZ/+*^ nuclei (green); scale bar: 20 μm. (**g**) Quantification of *Nrp1*^*lacZ/+*^ cells at the tip, normalized to overall contribution of cells to the endothelium; *n*=6 (number of retinas analysed); *P*<0.0001. (**h**–**k**) P5 retinas of *Nrp1*^*fl/fl*^*; mTmG; Cdh5-CreERT2* mice injected with 30 μg tamoxifen at P1, and treated with 100 mg kg^−1^ DAPT at P4. Unrecombined wt cells are labelled with Isolectin-B4 only; recombined *Nrp1*-deficient cells express GFP. (**h**) Representative overview of MOCK control. (**i**) Representative overview of DAPT-treated animals; scale bar, 100 μm, magnification is shown in **j**; scale bar, 20 μm. (**k**) Quantification of recombined *Nrp1*-deficient cells at the tip, normalized to overall contribution of cells to the endothelium. Statistical significance was determined by comparing the proportion of Nrp1-deficient (green) cells at the tip with the total proportion of Nrp1-deficient (green) cells; *n*=3 (number of retinas analysed); *P*<0.0013. (**g**,**k**) Values represent mean±s.e.m. Statistical significance was assessed using a Student's unpaired *t*-test.

To test this prediction, we investigated whether Notch inhibition by DAPT, which leads to marked hypersprouting in wt retinas^5^,^35^,^36^,^37^, can restore sprouting in the Nrp1-deficient vasculature. Remarkably, the Nrp1-deficient retinal vasculature did not respond to DAPT treatment with hypersprouting nor did DAPT rescue the outgrowth or branching defects, demonstrating that Notch inhibition is not able to induce excessive tip cell formation in the absence of Nrp1 (Fig. 3a–d). Furthermore, DAPT treatment of mosaic retinas or chimeric EBs failed to restore the ability of heterozygous or homozygous cells to reach the tip position (Fig. 3e–k; Supplementary Fig. 4e–g). Thus, unlike *Vegfr2* heterozygous cells, which regain the tip position upon Notch inhibition^7^, *Nrp1* heterozygous and homozygous deficient cells stay confined to the stalk position.

**Figure 4:**
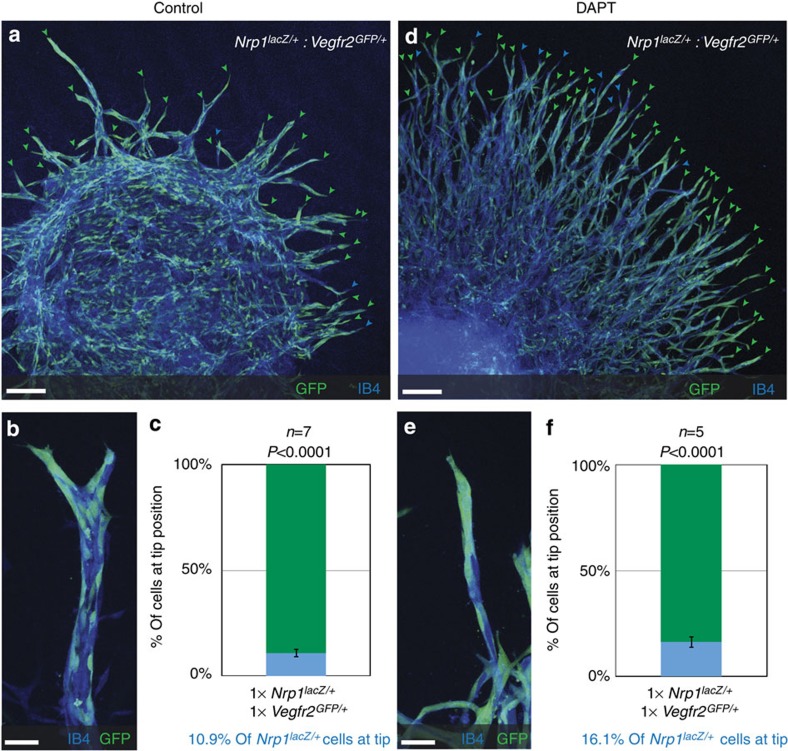
Nrp1 deficiency overrules Vegfr2 deficiency during tip cell competition. (**a**,**b**,**d**,**e**) Representative confocal images of the sprouting vasculature of a chimeric EB composed of *Vegfr2*^*GFP/+*^ cells and *Nrp1*^Lacz/+^ cells. Untreated (**a**,**b**) or treated with 5 μM DAPT for 5 days until harvesting at day 10 (**d**,**e**). (**a**,**d**) Tip cells derived from *Vegfr2*^*GFP/+*^ cells are indicated by green arrowheads and *Nrp1*^Lacz/+^ tip cells by blue arrowheads; scale bar, 130 μm. Magnifications of individual sprouts; scale bar, 36 μm (**b**,**e**). (**c**,**f**) Quantification of tip cells from *Vegfr2*^*GFP/+*^*:Nrp1*^*LacZ/+*^ chimeric EBs with equal input levels, either untreated (**c**) or treated with 5 μM DAPT (**f**). *n*=number of EBs analysed; number of counted tips: 458; *n*=7 (**c**) and 940; *n*=5 (**f**). *P* values were calculated using a Student's unpaired *t*-test by comparing quantified contribution with initial percentage of input levels; *P*<0.0001 (**c**,**f**). Values represent mean±s.e.m.

To directly determine whether Nrp1/Vegfr2 co-receptor activity is functionally involved in tip cell competition, we generated chimeric EBs mixing *Vegfr2* (ref. 7) and *Nrp1* heterozygous cells. If Vegfr2 co-receptor function accounted for tip cell formation, we expected equal handicap of *Vegfr2*^*+/−*^ and *Nrp1*^*+/−*^ cells to reach the tip. Surprisingly, we found that *Nrp1* heterozygous cells are not able to acquire the tip position even when competing against *Vegfr2* heterozygous cells (Fig. 4a–c). Again Notch inhibition was ineffective in restoring the balance at the tip (Fig. 4d–f). Thus a 50% gene dose reduction in Nrp1 impedes tip cell formation even when cells have twice the Vegfr2 level than their competing neighbours and when Notch is inactive. We conclude that unlike Vegfr2, Nrp1 does not operate upstream of Dll4/Notch and that Vegfr2 co-receptor function is unlikely to contribute to Nrp1 signalling events that regulate tip cell competition.

### Nrp1 affects Smad2/3 activation

**Figure 5:**
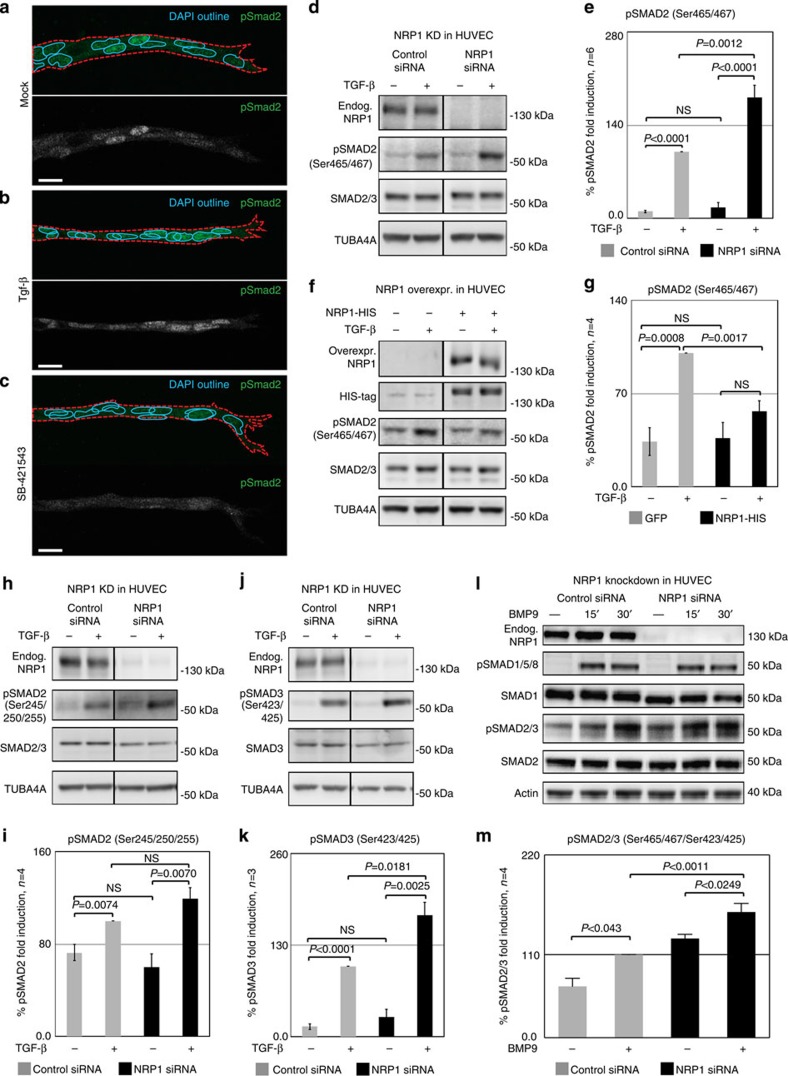
Nrp1 influences Smad2/3 activation. (**a**–**c**) Representative confocal images of wt sprouts from EBs immunolabelled for pSmad2. The outline of the wt sprouts is indicated with a dashed line using the endogenous DsRED marker. DAPI staining (not shown) was used to mark nuclei (blue line). Sprouts from (**a**) untreated EBs, (**b**) treated with 2 ng ml^−1^ Tgf-β for 1 h and(**c**) treated with 10 μM SB-421543 for 4 h. Scale bar, 13 μm. (**d**,**e**) Western blot analysis of proteins from P4 HUVEC transfected with control siRNA and NRP1 siRNA, with or without stimulation with 2 ng ml^−1^ TGF-β for 1 h. Full western blots are shown in Supplementary Fig. 10. A representative blot of six is shown; *P*=0.0012 NRP1 siRNA compared with control. (**f**,**g**) Proteins from P4 HUVEC transfected with control-GFP and NRP1–GFP–His construct for 24 h, with or without stimulation with 2 ng ml^−1^ TGF-β for 1 h were assessed for SMAD2 phosphorylation. Full western blots are shown in Supplementary Fig. 11. A representative blot of four is shown; *P*=0.0017 NRP1 overexpression compared with control. (**e**,**g**) Quantification of pSMAD2 protein normalized to SMAD2/3. (**h**–**k**) Western blot analysis of proteins from P4 HUVEC transfected with control siRNA and NRP1 siRNA, with or without stimulation with 2 ng ml^−1^ TGF-β for 1 h. Full western blots are shown in Supplementary Figs 12 and 13. (**i**) Quantification of pSMAD2 protein normalized to SMAD2/3; *P*=0.0848 Nrp1 siRNA compared with control. (**k**) Quantification of pSMAD3 protein normalized to SMAD3; *P*=0.0181 NRP1 siRNA compared with control. (**l**,**m**) Western blot analysis of P4 HUVEC transfected with control siRNA and NRP1 siRNA, with or without stimulation with 10 ng ml^−1^ BMP9 for 15 and 30 min. Full western blots are shown in Supplementary Fig. 14. A representative blot of four is shown. (**m**) Quantification of pSMAD2/3 protein normalized to SMAD2; *P*<0.0011 NRP1 siRNA compared with control. All values represent mean±s.e.m. DAPI, 4,6-diamidino-2-phenylindole; NS, not significant.

The Tgf-β pathway is known to inhibit sprouting, and Tgf-β has been shown to bind to Nrp1 in T cells and in cancer cells^16^,^17^,^20^,^38^. Its downstream effector Smad2 showed differential activation in EB sprouts, with strong nuclear staining in the stalk cells, but little or no pSmad2 in tip cells (Fig. 5a). Treatment with recombinant Tgf-β or the Alk5 inhibitor SB-431542 abolished the differential nuclear localization of pSmad2 (Fig. 5b,c).

To test whether Nrp1 influences Tgf-β-induced signaling, we measured Smad2 phosphorylation after stimulation with Tgf-β. Reduced Nrp1 expression in siRNA-treated HUVEC (Fig. 5d,e) or Nrp1-deficient EB (Supplementary Fig. 5a,b) consistently led to increased Smad2 phosphorylation. Conversely, overexpression of Nrp1 in HUVEC reduced pSmad2 levels compared with control-plasmid-transfected cells (Fig. 5f,g).

Phosphorylation of the Smad linker region (Ser 245/250/255), which is known to be induced after growth factor stimulation and might inhibit Smad activation, was unaffected by Nrp1 knockdown (Fig. 5h,i). However, C-terminal phosphorylation of Smad3 was also increased after Nrp1 knockdown (Fig. 5j,k), together indicating that Nrp1 levels quantitatively affect Tgf-β signalling via modulation of Smad2/3 phosphorylation.

Nrp1 knockdown also increased Smad2/3 activation in response to Bmp9, but did not affect phosphorylation of Smad1/5/8 in response to BMP9 or Tgf-β (Fig. 5l,m; Supplementary Fig. 5c–e). Thus, Nrp1 tonically suppresses Smad2/3 activation upon Tgf-β or Bmp9 stimulation.

**Figure 6:**
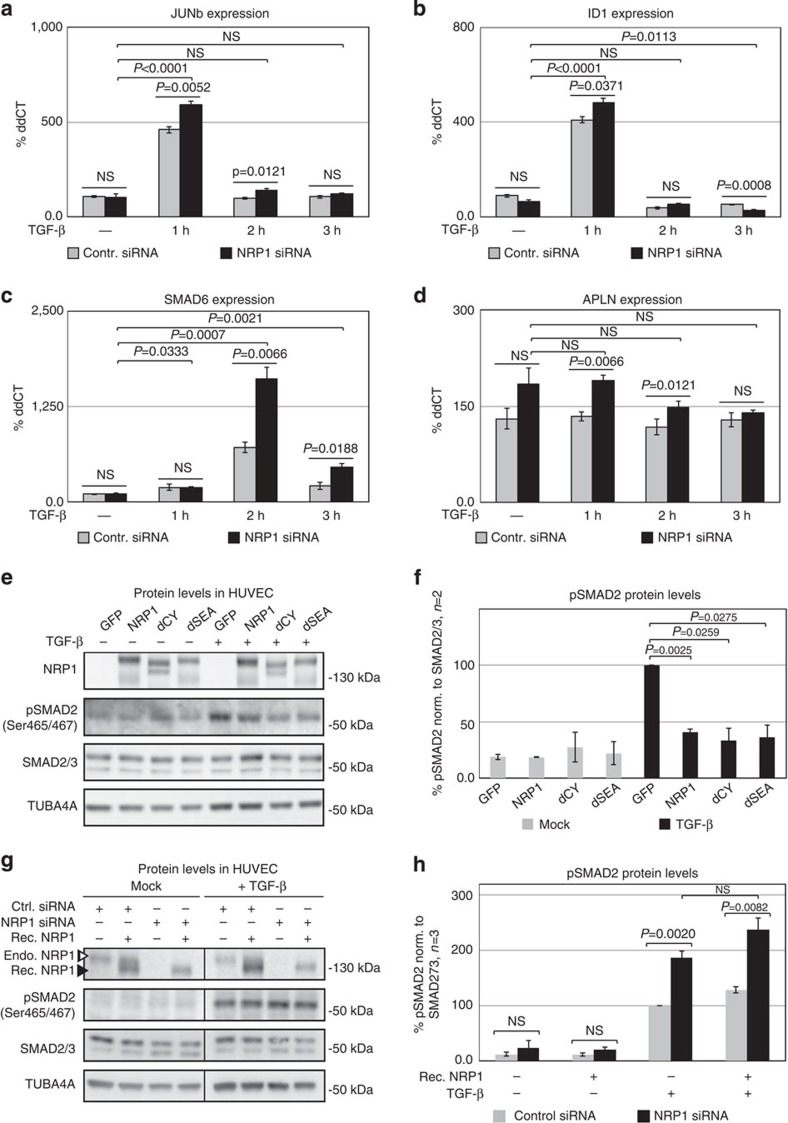
Nrp1 affects Tgf-β pathway activation independently of its intracellular domain. (**a**–**d**) Real-time quantitative PCR for JUNb, ID1, SMAD6 and APLN of P4 HUVEC transfected with control siRNA and NRP1 siRNA for 48 h. 2 ng ml^−1^ TGF-β stimulation was performed in a time course, 1, 2 and 3 h prior to cell lysis. All values are normalized to GAPDH and non-induced control siRNA. All samples have been performed as biological triplicates as well as technical triplicates; *P* values are indicated in the figure. (**e**,**f**) P4 HUVEC transfected with control-GFP construct, full-length NRP1–GFP–His construct, cytoplasmic-domain-deleted NRP1–dCY–GFP–His construct and SEA-domain-deleted NRP1–dSEA–GFP–His construct for 24 h prior stimulation with 2 ng ml^−1^ TGF-β for 1 h. Full western blots are shown in Supplementary Fig. 15. A representative of two biological repetitions is shown. (**f**) Quantification of pSMAD2 protein levels normalized to SMAD2/3; *P* values are indicated in the figure. (**g**,**h**) Western blot analysis of P4 HUVEC transfected with control siRNA and NRP1 siRNA for 48 h treated with 10 nM recombinant NRP1 1 h prior stimulation with 2 ng ml^−1^ TGF-β for 1 h. (**g**) Western blot for NRP1 shows endogenous (open arrowhead) and recombinant NRP1 (closed arrowhead). Full western blots are shown in Supplementary Fig. 16. A representative of three biological repetitions is shown. (**h**) Quantification of pSMAD2 protein normalized to SMAD2/3; *P* values are indicated in the figure. All values represent mean±s.e.m. Statistical significance was assessed using a Student's unpaired *t*-test. NS, not significant.

The expression of Smad target genes, including *JunB*, which can be activated through Tgf-β and Bmp2 (refs 39, 40, 41), *cJun*^42^, *Smad6* and *Smad7* (ref. 43), *Id1* (refs 44, 45), *Id3* (ref. 46), *Hey1* (refs 10, 11) and *Hes1* (refs 10, 11) were increased in HUVEC cells deficient for Nrp1, when stimulated with Tgf-β. In contrast, the expression of *Apln*, a gene that is upregulated in tip cells^47^, is not affected by the loss of Nrp1 when HUVEC cells were stimulated with Tgf-β (Fig. 6a–d; Supplementary Fig. 6a–e). Lack of Nrp1 also failed to affect Tgf-β or Bmp9 receptor expression (Supplementary Fig. 6f,g).

We next tested which domain of Nrp1 is critical for its effect on endothelial Smad activation. HUVECs transfected with constructs lacking either the C-terminal SEA domain that binds to PDZ adaptors^48^ or the entire cytoplasmic tail show a similar reduction of Tgf-β-induced Smad2 phosphorylation as the full-length construct (Fig. 6e,f). However, recombinant extracellular Nrp1 (ref. 49) did not reduce pSmad2 upon Tgf-β stimulation (Fig. 6g,h). We conclude that the extracellular and transmembrane domain of Nrp1 quantitatively regulates the endothelial response to Tgf-β.

### Alk5/Alk1 inhibition rescues sprouting in Nrp1-deficient cells

To investigate the functional impact of Alk/Smad2/3 signalling on sprouting behaviour and tip cell formation, we utilized a mosaic HUVEC *in vitro* tip cell competition assay^9^. When control siRNA- and *NRP1*-siRNA-transfected cells were mixed, more than 80% of tips were occupied by control siRNA-transfected cells (Supplementary Fig. 7a,b). In contrast, cells transfected with siRNAs against *ALK1*, *ALK5* or *SMAD2/3* preferentially occupy the tip position. Strikingly, simultaneous knockdown of *NRP1* together with *ALK1*, *ALK5* or *SMAD2/3* normalized tip cell contribution (Supplementary Fig. 7a,b). Treatment of mosaic HUVEC sprouting assays, chimeric EBs or mosaic *Nrp1*^*fl/fl*^; *mTmG; Cdh5-CreERT2* mice with Alk5 inhibitor SB-431542 restored the ability of Nrp1-deficient cells to reach the tip position (Supplementary Fig. 7c–j). SB-431542 also partially restored sprouting in Nrp1-deficient EBs (Supplementary Fig. 8).

**Figure 7:**
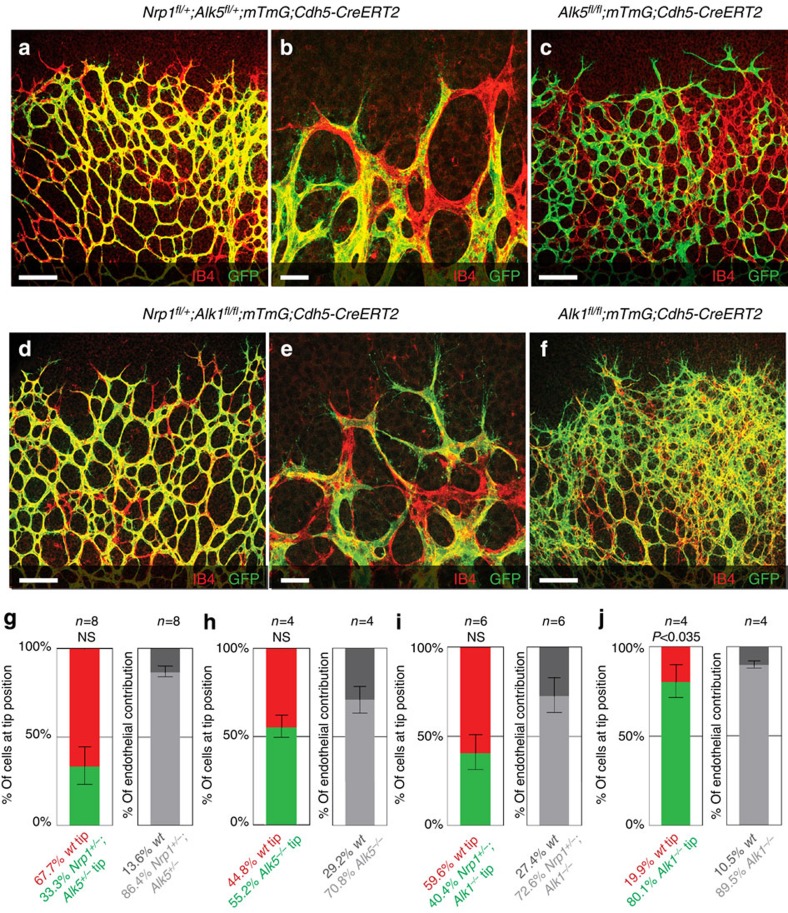
Inhibition of Alk5 and Alk1 rescues the Nrp1-deficient sprouting defect. Retinas of *Nrp1*^*fl/+*^*; Alk5*^*fl/+*^*; mTmG; Cdh5-CreERT2* mice (**a**,**b**), *Alk5*^*fl/fl*^*; mTmG; Cdh5-CreERT2* mice (**c**), *Nrp1*^*fl/+*^*; Alk1*^*fl/fl*^*; mTmG; Cdh5-CreERT2* mice (**d**,**e**) or *Alk1*^*fl/fl*^*; mTmG; Cdh5-CreERT2* mice (**f**) injected with 30 μg tamoxifen at P1; retinas were assayed P5. Unrecombined wt cells are labelled with Isolectin-B4 only; recombined cells express GFP. Magnification of **a** is shown in **b**, magnification of **d** is shown in **e**; scale bar, 100 μm (**a**,**c**,**d**,**f**), 20 μm (**b**,**e**). Quantification of recombined *Nrp1*^*fl/+*^*; Alk5*^*fl/+*^ (**g**), *Alk5*^*fl/fl*^ (**h**), *Nrp1*^*fl/+*^*; Alk1*^*fl/fl*^ (**i**) or *Alk1*^*fl/fl*^ (**j**) cells at the tip, normalized to overall contribution of cells to the endothelium. Statistical significance was determined by comparing the proportion of deficient (green) cells at the tip with the total proportion of deficient (green) cells; *P*=NS (0.0536) (**g**), *P*=NS (0.7576) (**h**), *P*=NS (0.275) (**i**), *P*<0.0358 (**j**). *n*=number of retinas analysed; *n*=8 (**g**), *n*=4 (**h**), *n*=6 (**i**) and *n*=4 (**j**); values represent mean±s.e.m. Statistical significance was assessed using a Student's unpaired *t*-test. NS, not significant.

To directly investigate the interaction between Nrp1 and Alk1/Alk5 *in vivo*, we generated compound *Nrp1*^*fl/+*^*; Alk5*^*fl/+*^*; mTmG; Cdh5-CreERT2* mice. Upon low-dose tamoxifen treatment, 33.3% of *Nrp1/Alk5*-deficient cells were found at the tip position (Fig. 7a,b,g), as opposed to 11.9% of single-*Nrp1* heterozygote cells (Fig. 1i). Thus, deleting one copy of *Alk5* partially rescues the capacity of Nrp1 heterozygous cells to attain the tip position. Interestingly, however, Alk5 deficiency alone in mosaic *Alk5*^*fl/fl*^*; mTmG; Cdh5-CreERT2* retinas did not promote preferential tip cell localization (Fig. 7c,h), indicating a strong and selective genetic interaction between Alk5 and Nrp1 in the process of tip cell selection.

To determine whether Bmp9-Alk1 signalling also contributed to the effects of Nrp1 inhibition *in vivo*, we generated *Nrp1*^*fl/+*^*; Alk1*^*fl/fl*^*; mTmG; Cdh5-CreERT2* mice. Upon low-dose tamoxifen treatment, 28.1% of *Nrp1/Alk1*-deficient cells were found at the tip position (Fig. 7d,e,i), as opposed to 80.1% single-*Alk1* homozygous cells (Fig. 7f,j). In addition, hypervascularization seen in endothelial *Alk1*^*fl/fl*^ mutants appeared strikingly normalized by deletion of one allele of *Nrp1*. Thus, while single-Alk1- and Nrp1-deficient cells show strong preference for tip and stalk, respectively, reducing Nrp1 levels in Alk1 mutants by 50% efficiently normalizes tip cell contribution and vascular patterning.

## Discussion

In summary, our mosaic studies reveal that the loss of only one allele of Nrp1 renders cells unable to compete with wt cells for the tip cell position, in the absence of major vascular phenotypes in heterozygous mice. Importantly, mosaic *in vivo* deletion by induced Cre activation and blastocyst injections to create chimerism that does not rely on Cre reporters both independently validated the principal finding that Nrp1 heterozygous cells are not able to compete for the tip. Further validation was achieved using the EB system and in HUVEC spheroids, two more reductionist models that lack system contributions. Remarkably, Nrp1-deficient cells are unable to attain the tip position even when Notch is inhibited, and the loss of Nrp1 renders the vasculature completely refractory to the loss of Notch. Notch inhibition normalizes tip cell contribution in ECs deficient for Vegfr1, 2 or 3 (refs 7, 8), but not in Nrp1-deficient cells, indicating that in contrast to Vegfrs, including Vegfr2, that act upstream of Dll4/Notch^50^, Nrp1 operates downstream of Notch. Given that Nrp1 is quantitatively regulated by Notch activity^51^, these results identify Nrp1as the critical downstream effector of Notch in tip/stalk specification during angiogenesis.

Our finding that Nrp1 has functions beyond acting as a Vegfr2 co-receptor could explain why knock-in mice expressing an Nrp1 variant that cannot bind Vegf are born at Mendelian ratios and show only minor embryonic angiogenesis defects, while global Nrp1 knockouts are embryonic lethal due to severely disrupted vasculature^52^,^53^. In addition, our data show that any Vegfr2 surface regulation by Nrp1 does not affect Dll4 levels and therefore does not operate upstream of Notch signalling in tip cell selection. Blocking of Vegf binding to Nrp1 using a specific antibody that leaves Vegf binding to Vegfr2 intact^54^ does not phenocopy the retinal angiogenesis defects in Nrp1 mutants described here, and Nrp1 promotes activation of intracellular c-abl activation in a VEGF-independent manner^55^, further supporting the idea that Nrp1 may affect other pathways that trigger tip cell formation.

**Figure 8:**
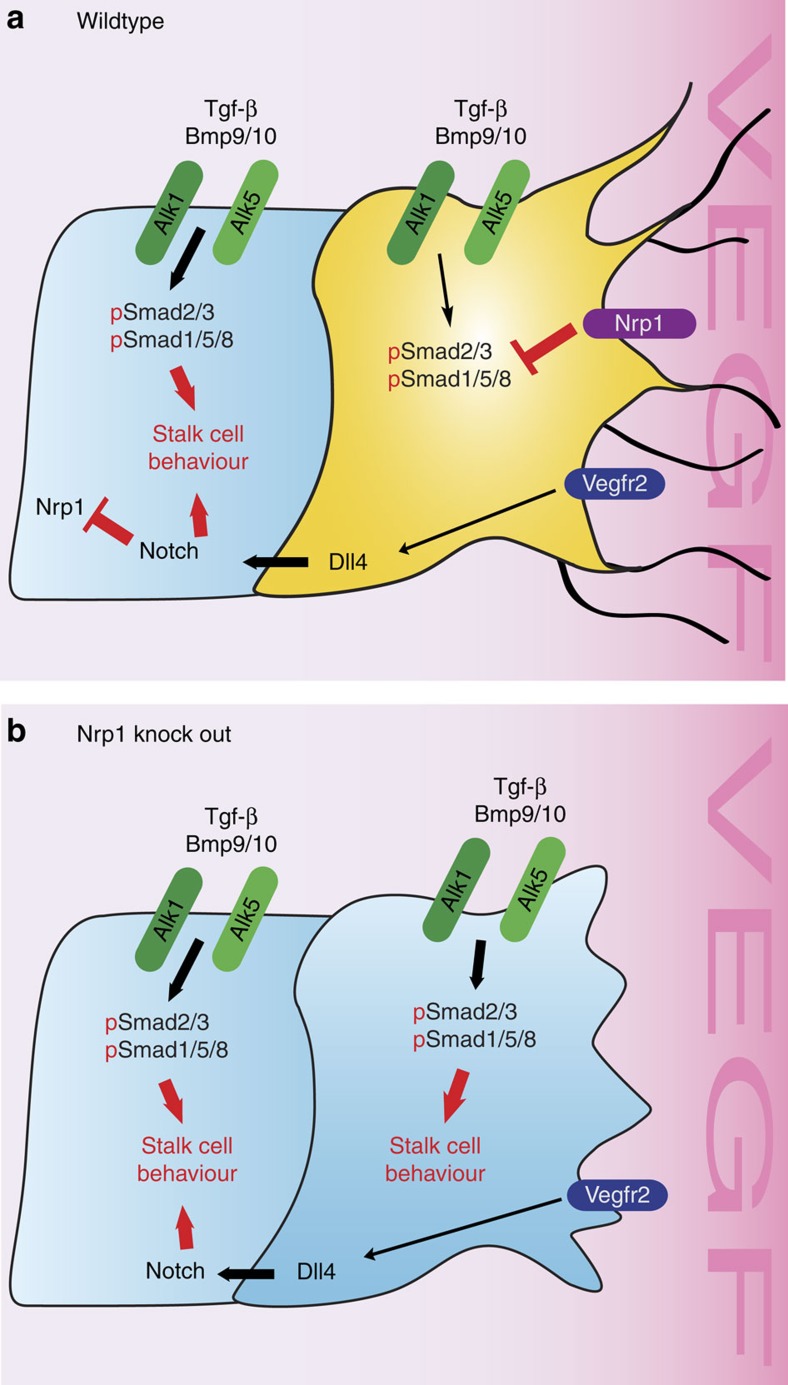
Working model for Nrp1 function in tip cell formation. (**a**) The data suggest that Nrp1 inhibits Smad2/3 phosphorylation by Alk5 and Alk1 in response to Tgf-β and Bmp9/10 in tip cells (yellow). VEGF-dependent, but Nrp1-independent upregulation of Dll4 in the tip leads to Notch activation in stalk cells, which decreases Nrp1 levels in the stalk cell (blue). The reduction of Nrp1 in the stalk results in higher pSmad2/3 levels and stalk cell behaviour. (**b**) In an *Nrp1* knock-out situation, pSmad2/3 activation leads to the activation of stalk cell behaviour in all endothelial cells, resulting in sprouting inhibition.

Our results identify a significant overactivation of the Smad2/3 pathway in Nrp1-deficient cells as the key determinant of loss of tip cell capacity and sprouting. They are consistent with a model in which Notch activation in stalk cells decreases Nrp1 levels, which induces stalk cell behaviour by suppressing Smad2/3 activation downstream of Alk1 and Alk5. High levels of Nrp1 suppress Smad2/3 activation in wt tip cells, while increased Alk1/Alk5 signalling in Nrp1 mutant endothelial cells leads to inhibition of tip cell formation (Fig. 8).

Tgf-β has well-known effects on vessel sprouting and stability^56^,^57^, but has not been implicated directly in tip/stalk specification. Previous work established that Tgf-β addition to embryonic stem (ES) cells cultures blunts sprouting^17^. Whereas the balance between Alk1 and Alk5 signalling has been shown to regulate vascular function^14^, and Alk1-mediated Smad signalling was shown to co-operate with Notch to induce the stalk cell fate^10^,^11^, the relative contributions of Smad1/5/8 and Smad2/3 in tip/stalk cell specification are not fully understood. pSmad2 staining in the EB system preferentially highlighted stalk cell nuclei, providing first indications for differential activity. Tgf-β stimulation, which curbed sprouting, lead to high pSmad2 levels in all endothelial cells, an effect that was sensitive to the Alk5 inhibitor SB-431542. Together with the observed hypersprouting phenotype induced by SB-431542, these results suggest that endogenous Tgf-β signalling drives differential pSmad2 activity via Alk5 in the endothelium.

Given that we observed little effect of Nrp1 on pSmad1/5/8, we initially suspected that the prominent role of Bmp/Alk1 in tip/stalk specification^11^ would be distinct and not influenced by Nrp1. However, endothelial cells responded with Smad2/3 phosphorylation even when stimulated with Bmp9, and showed increased pSmad2/3 in the absence of Nrp1 also under Bmp9 stimulation. These data suggest that tip cell Nrp1 inhibits Bmp9-Alk1 signalling and implicates Smad2/3 signalling in this response. The identification of Nrp1 as a modulator of Alk1 signalling output, and the striking normalization of the hypervascular phenotype in Alk1-deficient retinas by deletion of a single copy of Nrp1 raises the prospect that targeting Nrp1 activity could provide treatment options for Hereditary Haemorrhagic Telangiectasia (HHT) patients carrying inactivating Alk1 mutations^58^.

How Nrp1 inhibits Smad2/3 signalling remains to be determined. Nrp1 has been shown to bind latent Tgf-β and to interact with Alk5 in tumour cells and fibroblasts^59^,^60^, suggesting that Nrp1 might act as a ligand trap in endothelial cells. However, the fact that soluble Nrp1 fails to inhibit Tgf-β-induced Smad2/3 activation argues against this possibility. Nrp1 might act as a decoy by virtue of its extracellular or transmembrane domains, which may inhibit interactions between type 1 and type 2 receptors and Smad phosphorylation. We show that phosphorylation of the Smad linker region, which negatively modulates Smad signalling^61^, is unaffected by Nrp1. Decreased Nrp1 also failed to affect expression levels of endothelial type 1 or type 2 receptors, or the co-receptor endoglin (Eng), although it cannot be excluded that other circulating ligand traps are modulated by Nrp1. Further studies are required to elucidate the mechanistic basis for Nrp1 suppression of Smad2/3 signalling and its interaction with Alk1 and Alk5.

Conceptually, these results radically change the view of Notch function and tip cell formation. Whereas the prevailing paradigm presumes that Notch is the critical brake on angiogenesis, as blocking of Dll4/Notch even in the adult is sufficient to activate new sprouting and tip cell formation, our results indicate that instead Smad2/3 signalling is the major constraint on sprouting and tip cell formation. Notch takes a role as a modulator, establishing through lateral-inhibition heterogeneity in the Tgf-β/Bmp–Smad2/3 response. Notch patterns the differential Smad2/3 response by regulating Nrp1, thus allowing tip/stalk specification and regular sprouting. Implicit in this view is a second conceptual shift: the tip cell is not just the default response, but requires active suppression of the stalk cell phenotype.

This change in our understanding of the principal mechanisms regulating endothelial responses has wide implications for therapy, as Nrp1 inhibition becomes a highly attractive target to curb angiogenesis, or to modulate Tgf-β/Bmp responses in vascular malformations.

## Methods

### Mice

Mice for blastocyst injection were maintained at the London Research Institute. Animal procedures were performed in accordance with the Home Office Animal Act 1986 under the authority of project license PPL 80/2391. Mice for inducible gene deletion were maintained in the Animal Research Center at Yale University and experiments were approved by the IACUC of Yale University. The following mouse strains were used: *Tg(CAG-DsRED-MST)Nagy/J* (Jackson Laboratory, USA)^62^, were used as hosts to generate chimeric retinas. *C57/Bl6, mT/mG*^34^ and *Nrp1*^*flox*^^31^ have been described previously and were obtained from Jackson Laboratories. *Cdh5-CreERT2* mice^32^,^33^ were provided by Ralf Adams. *Alk1*^*flox*^ and *Alk5*^*flox*^ mice^63^,^64^ were kindly provided by Paul Oh and Stefan Karlsson, respectively.

### Inducible gene deletion

Cre activity and gene deletion were induced by intraperitoneal injections to male and female pups with 100 μg tamoxifen (Sigma, T5648; 2 mg ml^−1^) at postnatal day (P) 1, P2 and mice were killed at P5. 30μg tamoxifen was injected at P1 to induce mosaic deletion and mice were killed at P5.

### Animal procedures

Embryonic stem cells in suspension (see Mouse ES Cells section) were injected into 3.5-day post-coitum-stage embryos produced by mating homozygote Tg(CAG-DsRED*MST)1Nagy/J mice to *C57BL/6J* mice according to standard protocols after which embryos were reimplanted into pseudopregnant *C57BL/6J* foster mice^65^. Mice were killed at P5 for analysis of retinal vasculature.

DAPT (Sigma, D5942) was subcutaneously injected to male and female pups at P4 using 20 μl g^−1^ of 5 mg ml^−1^ working solution and mice were killed 24 h later. For EdU staining (Life Technologies, A10044), male and female mice were intraperitoneally injected with 20 μl g^−1^ of 0.5 mg ml^−1^ of EdU at P5. Harvest of the eyes was performed 2 h after EdU injection. Twenty mg kg^−1^ SB-431542 (Sigma, S4317; 10 mg ml^−1^) was intraperitoneally injected in male and female pups at P3 and P4, and mice were killed at P5.

### Retina analysis and quantification

Eyes were harvested at P5 and fixed with 4% paraformaldehyde on ice for 2 h, or at room temperature for 18 min, followed by retinal dissection before staining was performed. Whole mount was performed using VectaShield mounting media (Vector Laboratories).

High-resolution three-dimensional rendering of retinas and embryoid bodies were acquired using a Laser Scanning Microscope 780 confocal system (Zeiss), using the ZEN 2010B SP1 software, version 6.0 (Zeiss) or a Leica SP5 confocal microscope using a Leica spectral detection system (Leica SP detector) and the Leica application suite advanced fluorescence software.

### Mouse ES cells

Mouse ES cells expressing DsRED-MST^62^ were a kind gift from A. Nagy and J. Rossant (Samuel Lunenfeld Research Institute, Canada). *Vegfr2*^*+/egfp*^ ES cells were kindly provided by A. Medvinsky (Edinburgh, UK)^7^. *Nrp1*^*Lacz/+*^ and *Nrp1*^*Lacz/LacZ*^ cells were generated by replacing exons 1 and 2 with a LacZ cassette through homologous recombination^66^ (Regeneron Pharmaceuticals).

### Embryoid bodies (EBs)

Culturing of mouse ES cells and generation of EBs was performed as described below and as previously described^67^. ES cell clones were cultured in DMEM/glutamax (Invitrogen), 25 mM HEPES, 1.2 mM sodium pyruvate, 19 mM monothioglycerol (Sigma), 15% fetal bovine serum and 1,000 units per ml leukaemia inhibitory factor on gamma-irradiated feeder cells. Different genotypes were mixed as indicated in the individual figures and left in suspension as hanging drops without the leukaemia inhibitory factor (Millipore, ESG1107) (day 0). After 5 days, the formed spheroids were embedded in a polymerized collagen I gel and stimulated with 30 ng ml^−1^ VEGFA165 (Peprotech, 450-32) to grow in a three-dimensional fashion. The collagen solution was prepared using 50% 3 mg ml^−1^ collagen I (Purecol), 34.65% 1 × F-12 Nutrient Mix (Gibco), 0.625% 100 × Glutamax (Gibco), 0.977% sodium bicarbonate 7.5% (Gibco), 1.25% 1 M HEPES (Gibco), 6.25% 10 × F-12 Nutrient Mix (made in house) and 6.25% 0.1 N NaOH (Sigma).

Medium with dimethylsulphoxide (Sigma, D4540), 5 μM DAPT (Sigma, D5942), 2 ng ml^−1^ TGF-β (PeproTech, 100-21C) or 10 mM SB-431542 (Tocris, 1614) was changed on day 6 and every day thereafter. At day 10, EBs were fixed in 4% paraformaldehyde at room temperature for 15 min.

EBs used for protein and RNA extraction were grown on six-well plates incubated with 0.2% gelatine (Sigma, G1393) after 5 days of hanging drops and stimulated with 30 ng ml^−1^ VEGFA165 (Peprotech, 450-32). Samples were harvested after 4 days at day 9 using the respective lysis buffer for RNA or protein extraction.

### Antibodies

For immunostaining and western blotting (WB), the following antibodies were used: goat anti-mouse Nrp1 (1:500; R&D, AF566), rabbit anti-human Nrp1 (1:1,000, Cell Signaling, 3,725), mouse anti-Smad2/3 (1:500; BD, 610,843), rabbit anti-pSmad2 (Ser465/467) (1:500; Millipore, 04-953), rabbit anti-pSmad2/3 (Ser465/467 Ser423/425) (1:1,000; Cell Signaling, 9,510), rabbit anti-pSmad2 (Ser245/250/255) (1:1,000; Cell Signaling, 3,140), rabbit anti-pSmad3 (Ser423/425) (1:500; Abcam, ab52903), rabbit anti-Smad3 (1:500; Abcam, ab40854), rabbit anti-pSmad1/5/8 (Ser463/465 Ser426/428) (1:1,000; Cell Signaling, 9,511), rabbit anti-Smad1 (1:1,000; Cell Signaling, 9743), rat anti-Pecam antibody (1:50; BD Pharmingen, 550274), goat anti-mouse Dll4 (1:200 for immunohistochemistry analysis and 1:500 for WB; R&D, AF1389), rabbit anti-Erg1/2/3 (1:200, Santa Cruz, sc-353), Nicd (1:500, Cell Signaling, 2421), mouse anti-α-tubulin (1:5000, Sigma, T5168), mouse anti-HIS-tag (1:1,000, Abcam, ab18184) and rabbit anti-GFP (1:1,000; Invitrogen, G10362).

Secondary antibodies: Alexa-488 donkey anti-goat IgG (A11055), Alexa-488 donkey anti-rabbit (A21206), Alexa-647 donkey anti-rabbit IgG (A31573), isolectin-B4 directly conjugated to Alexa-488 (I21411), all diluted 1:500, obtained from Life Technologies. Alexa-647 donkey anti-rat (1:500, Stratech Scientific, 712-606-153-JIR). Horseradish peroxidase (HRP)-conjugated anti-rabbit IgG (1:8,000; GE-Healthcare; NA3940), HRP-conjugated anti-mouse IgG and HRP-conjugated, (1:8,000; GE-Healthcare; NA931V). Rabbit anti-goat IgG (1:2,000; Dako; P0449).

### Western blotting

Cells were lysed in D0.4 lysis buffer including phosphatase and protease inhibitors (Thermo Scientific, 78420, 1862209). BCA protein assay (Fisher, 13276818) was used to measure the protein concentration. Equal amounts of proteins were separated on NuPAGE 4–12% MOPS gel (Life Technologies, NP0336) and transferred on polyvinylidene difluoride membrane (GE Healthcare Biosciences, RPN2020F). WBs were developed with chemiluminescence HRP substrate (Millipore, WBKLS0500) on a Luminescent image analyser, ImageQuant LAS 4000 mini (GE Healthcare). Band intensity was measured using ImageQuant TL 1D, version 7.0.

### Quantitative real-time PCR

Primary endothelial cells were isolated from mouse lung by digestion of lung tissue with gentle agitation at 37 °C in PBS with 2 mg ml^−1^ type 1 collagenase (Sigma), followed by filtration through a 70-μm disposable cell strainer (Falcon). Endothelial cells were isolated using rat-anti-mouse Dynabeads (Life Technologies) coated with anti-PECAM antibody, according to manufacturer's instructions. RNA from HUVEC or from primary endothelial cells isolated from lung^68^ was extracted and purified using RNeasy-kit (Qiagen). RNA concentrations were measured on a Nanodrop and adjusted equally, followed by reverse transcription by SuperScript III (Invitrogen). Quantitative PCR with Taqman probes for m*Dll4* (Mm00444619), m*Cd31* (Mm01242584), m*Nrp1* (Mm00435379), m*Notch1* (Mm00435245), m*Hey1* (Mm00468865), m*Alk1* (Mm_Acvrl1_1_SG QT00161434), m*Alk2* (Mm_Acvr1_1_SG QT00093422), m*Alk3* (Mm_Bmpr1a_1_SG QT01057511), m*Alk4* (Mm_Acvr1b_1_SG QT00163464), m*Alk5* (Mm_Tgfbr1_1_SG QT00135828), m*Alk6* (Mm_Bmpr1b_1_SG QT00121240), m*Alk7* (Mm_Acvr1c_1_SG QT00262591), m*BmpR2* (Mm_Bmpr2_1_SG QT00251349), m*TgfBR2* (Mm_Tgfbr2_1_SG QT00135646), m*Eng* (Mm_Eng_1_SG QT00148981), h*SMAD6* (Hs00178579), h*HES1* (Hs00172878), h*ID1* (Hs03676575), h*APLN* (Hs00178579), h*HEY1* (Hs01114113), h*ID3* (Hs00171409), GAPDH (4326317E), h*NRP1* (Hs00826128), h*ALK1* (Hs_Acvrl1_1_SG QT00050351), h*ALK2* (Hs_Acvr1_1_SG QT00071743), h*ALK3* (HS_Bmpr1a_1_SG QT00085358), h*ALK4* (Hs_Acvr1b_1_SG QT00053235), h*ALK5* (Hs_Tgfbr1_1_SG QT00083412), h*ALK6* (Hs_Bmpr1b_1_SG QT00084469), h*Alk7* (Hs_Acvr1c_1_SG QT00042819), h*BMPR2* (Hs_Bmpr2_1_SG QT00226065), h*TGFBR2* (Hs_Tgfbr2_1_SG QT00014350) and h*ENG* (Hs_Eng_1_SG QT00013335) were run in an Applied Biosystems 7900HT real-time thermal cycler. The expression levels were normalized to Pecam for EBs and to GAPDH for HUVECs.

### Sprouting assay

HUVECs were transfected with 25 pmol siRNA (FlexiTube siRNA, Qiagen) per well of a six-well plate using 2.5 ml RNAiMax (Invitrogen) according to the manufacturer's instructions. After 24 h, HUVECs were labelled with either PKH26 (red) or PKH67 (green) (Sigma-Aldrich) according to the manufacturer's instructions, and a ratio of 1:1 cells were coated on cytodex3 microcarrier beads (Sigma-Aldrich) for 24 h. Beads were embedded in a fibrin gel (2.5 mg ml^−1^ fibrinogen (Sigma-Aldrich) in EBM-2 (Lonza) supplemented with 2% FBS and 50 mg ml^−1^ aprotinin (Sigma-Aldrich)) with fibrinogen solution clotted with 1 U thrombin (Sigma-Aldrich) for 30 min at 37 °C. WI38 cells (25,000 cells per well), in EBM-2 supplemented with 2% FBS and 50 ng ml^−1^ VEGF, were then plated on top of the fibrin layer. Sprouts were imaged 3–4 days later on a Nikon eclipse Ti confocal microscope with the PerkinElmer UltraVIEW Confocal Imaging System and PerkinElmer Volocity software.

### Cell culture experiments

HUVECs (PromoCell) were cultured in EGM2-Bulletkit (Lonza), following the manufacturer's manual, and used for all experiments at passage 2–4. SiRNA transfection for *NRP1* (J-019484-06), *VEGFR2* (J-003148-09) and control siRNA (D-001810-01), *ALK1* (#M-005302-02), *ALK5* (#M-003929-02), *SMAD2* (#M-003561-01) and *SMAD3* (#M-020067-00), all obtained from Dharmacon, was performed 48 h prior harvesting using Dharmafect 1 (Dharmacon), following the provided manual. NRP1 plasmids (pEGFP-N1_mNRP1, pEGFP-N1_mNRP1dSEA and pEGFP-N1_mNRP1dCY) have been a kind gift from Dr Guido Serini (IRCC, Institute for Cancer Research and Treatment and Department of Oncological Sciences, University of Torino School of Medicine). Transient transfection was performed using Lipofectamin 2000 (Invitrogen) for 24 h prior harvesting. Cells were treated with 10 mM recombinant NRP1 (R&D, 5994-N1), 2 ng ml^−1^ TGF-β (R&D and PeproTech), SB-431542 (Sigma and Tocris), 10 ng ml^−1^ BMP9 (R&D) or 5 μM DAPT (Calbiochem and Sigma).

### Image segmentation

Confocal images of chimeric retinas obtained from blastocyst injection were processed in Imaris 7.6.1 (Bitplane). Using the endothelial marker Pecam, a surface mask was created to subtract endothelial cells from background signal. Within the Pecam mask a second surface mask was generated using the DsRED signal expressed by wt cells. The DsRED mask was used to separate Erg-positive nuclei within the DsRED mask from Erg-positive nuclei outside the DsRED mask but inside the Pecam mask. Using pseudo-colours both sets of Erg signals were highlighted in different colours as indicated in the figure.

### Statistical analysis and image processing

Student's unpaired *t*-tests for comparison were performed for all quantitative data, if not indicated differently, using Prism 6.0 (Graph Pad), Excel (Microsoft). Image processing was performed using Imaris 7.6.1 (Bitplane), Volocity 5.5 (Perkin Elmer) and Photoshop CS5 (Adobe), in compliance with ‘Nature Press Data Processing Policy'.

## Additional information

**How to cite this article:** Aspalter, I. M. *et al.* Alk1 and Alk5 inhibition by Nrp1 controls vascular sprouting downstream of Notch. *Nat. Commun.* 6:7264 doi: 10.1038/ncomms8264 (2015).

## Supplementary Material

Supplementary Information Supplementary Figures 1-20
