# Supplementary material for: Alk1 and Alk5 inhibition by Nrp1 controls vascular sprouting downstream of Notch
Source: Nat Commun. 2015 Jun 17;6:7264. doi: 10.1038/ncomms8264 (PMC4557308; doi:10.1038/ncomms8264)
Supplement: Supplementary Information — Supplementary Figures 1-20 [file ncomms8264-s1.pdf]

Supplementary Figure 1

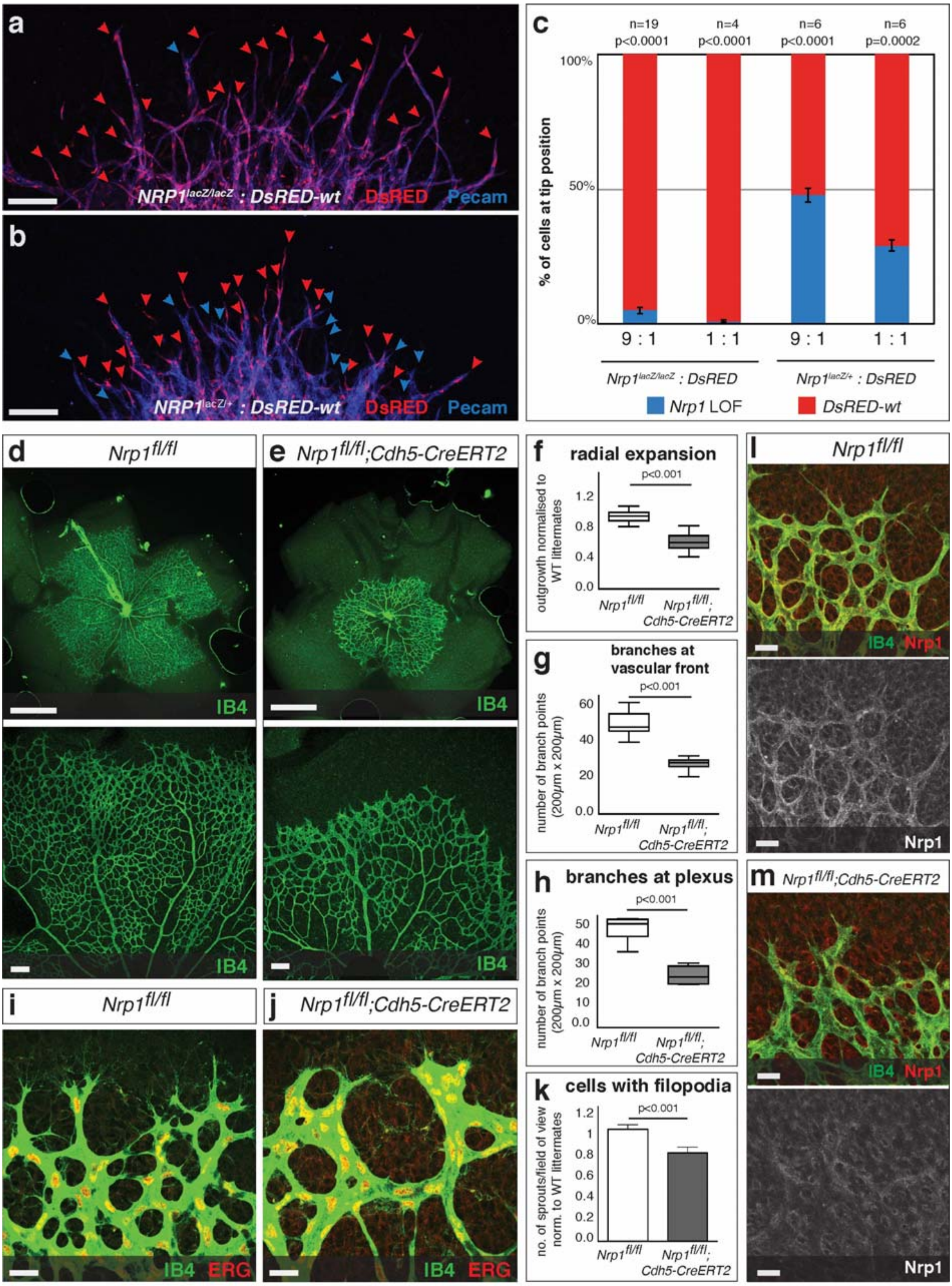

Supplementary Figure 1: Nrp1 deficient mice show severe sprouting defects in retinal vessels

(a, b) Representative confocal images of the sprouting vasculature of a chimeric EB composed of wt cells and Nrp1 deficient cells. Blue arrowheads indicate Nrp1 deficient tip cells, red arrowheads highlight wt tips. (a) *Nrp1*$^{lacZ/lacZ}$ cells and (b) *Nrp1*$^{lacZ/+}$ cells mixed in a 9:1 ratio with wt cells. Scale bar a, b: 160µm (c) Quantification of tip cells; n = EBs per condition; n = 19 (593 tips counted in total) for *DsRED:Nrp1*$^{LacZ/LacZ}$ = 1:9, p<0.0001; n = 4 (734 tips counted) for *DsRED:Nrp1*$^{LacZ/LacZ}$ = 1:1, p<0.0001; n = 6 (504 tips counted) for *DsRED:Nrp1*$^{LacZ/+}$ = 1:9, p<0.0001; n = 6 (314 tips counted) for *DsRED:Nrp1*$^{LacZ/+}$ = 1:1, p<0.0002 □. P-values were calculated using Student's unpaired t-test by comparing quantified contribution to initial percentage of input levels. Values represent mean ± s.e.m.

(d, e) IsolectinB4-stained P5 retinal vasculature of *Nrp1*$^{fl/fl}$ and *Nrp1*$^{fl/fl}$*;Cdh5-CreERT2* mice, injected with 100µg tamoxifen at P1 and P2. Representative overview of retinal vessels (d, e), scale bar: 200µm, and higher magnification of one petal (lower panel) of (d) *Nrp1*$^{fl/fl}$ mice and (e) *Nrp1*$^{fl/fl}$*; Cdh5-CreERT2* mice, scale bar: 200µm. (f) Quantification of radial expansion normalized to wt littermates, p<0.001, (g) branch points at the vascular front, p<0.001, and (h) branch points at the plexus, p<0.001. For all quantifications n>8 (where n represents the number of retinas), values represent mean ± s.e.m. (i, j) Magnification of the sprouting front of *Nrp1*$^{fl/fl}$ and *Nrp1*$^{fl/fl}$*; Cdh5-CreERT2* mice labeled with Isolectin-B4 (green) and Erg (red), scale bar: 20µm. (k) Quantification of cells with filopodial extensions, n=5 (where n represents the number of retinas), p<0.001, values represent mean ± s.e.m. P-values were calculated using Student's unpaired t-test.

(l, m) Magnification of the sprouting front of *Nrp1*$^{fl/fl}$ and *Nrp1*$^{fl/fl}$*; Cdh5-CreERT2* mice labeled with Isolectin-B4 (green) and Nrp1 (red). Note absence of endothelial Nrp1 staining in *Nrp1*$^{fl/fl}$*; Cdh5-CreERT2* retinas (m), while non-endothelial staining is not affected. Scale bar: 20µm.

Supplementary Figure 2

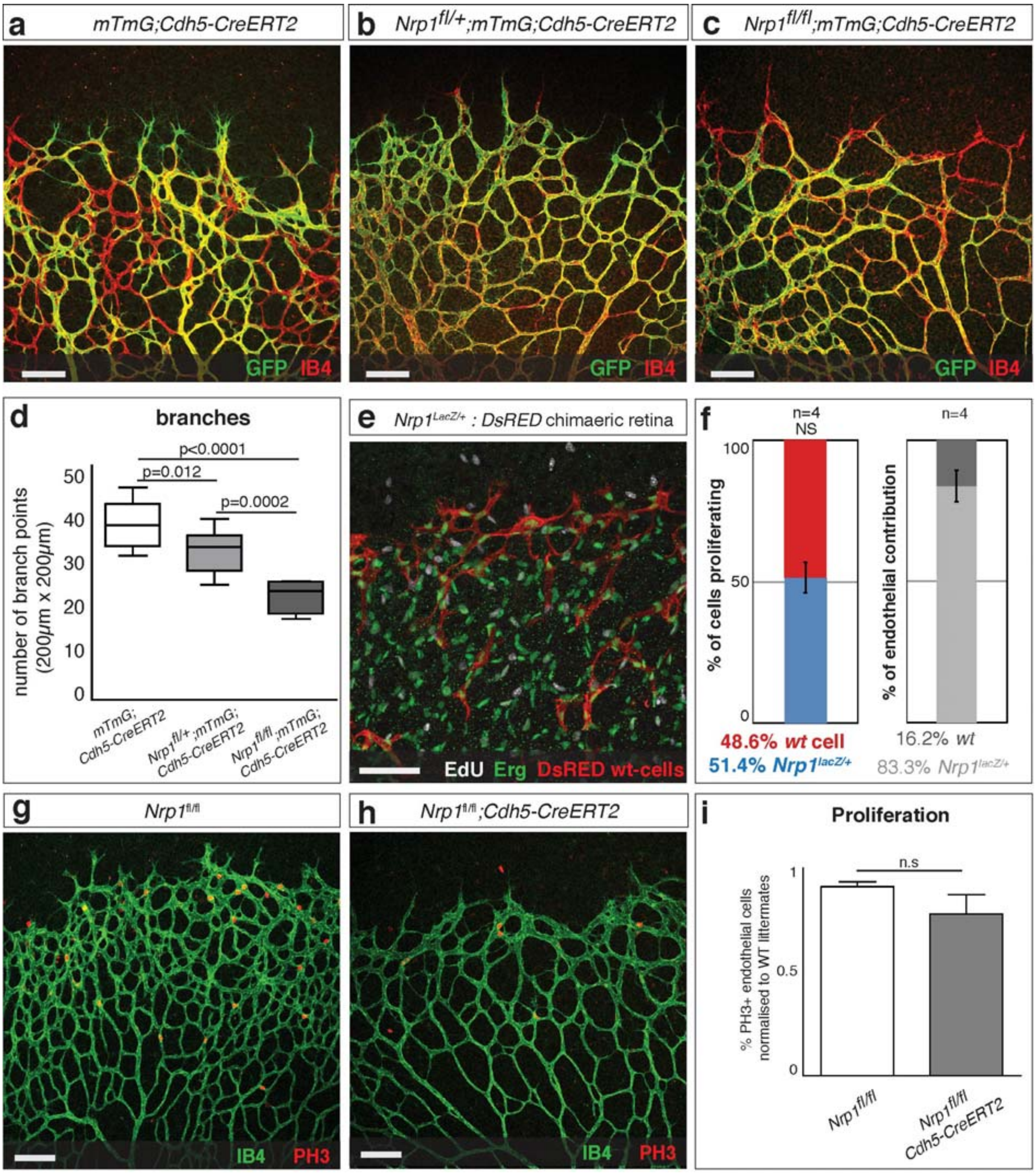

Supplementary Figure 2: Vascular patterning defects following Nrp1 deletion

(a-c) Retinas of *mTmG; Cdh5-CreERT2, Nrp1$^{fl/fl}$; mTmG; Cdh5-CreERT2* and *Nrp1$^{fl/+}$; mTmG; Cdh5-CreERT2* mice injected with 30µg tamoxifen at P5, retinas were assayed P5. Scale bar: 100µm. (d) Quantification of branch points. n = the number of retinas analyzed; n>6. P values are indicated in the figure.

(e) Retinal vessels from wild-type host expressing DsRED injected with *Nrp1$^{Lacz/+}$* ES cells, treated with EdU. Mice were intraperitoneally injected with 20ul/g of 0.5 mg/ml of EdU at P5. Eyes were harvested 2h after EdU injection. Scale bar: 80µm. (f)

Quantification of the numbers of proliferating $Nrp1^{Lacz/+}$ cells and proliferating DsRED-wt cells, normalized to the endothelial contribution of each genotype to the overall vasculature. n = 4 (numbers of retinas); no statistical difference between wt and $Nrp1^{Lacz/+}$ cells. (g-i) $Nrp1^{fl/fl}$ and $Nrp1^{fl/fl}$;*Cdh5-CreERT2* mice, injected with 100µg tamoxifen at P1 and P2, analyzed at P5. Dividing cells were visualized using PH3 immunostaining. Scale bar: 100µm. (i) Quantification of the number of dividing PH3+IsoB4+ cells, normalized to the proportion of IsoB4 endothelial cells, and normalized to wt littermates. n = 3 (the number of retinas analyzed); no statistical difference between $Nrp1^{fl/fl}$ and $Nrp1^{fl/fl}$;*Cdh5-CreERT2 cells*. All values represent mean ± s.e.m. All p-values were calculated using Student's unpaired t-test.

Supplementary Figure 3

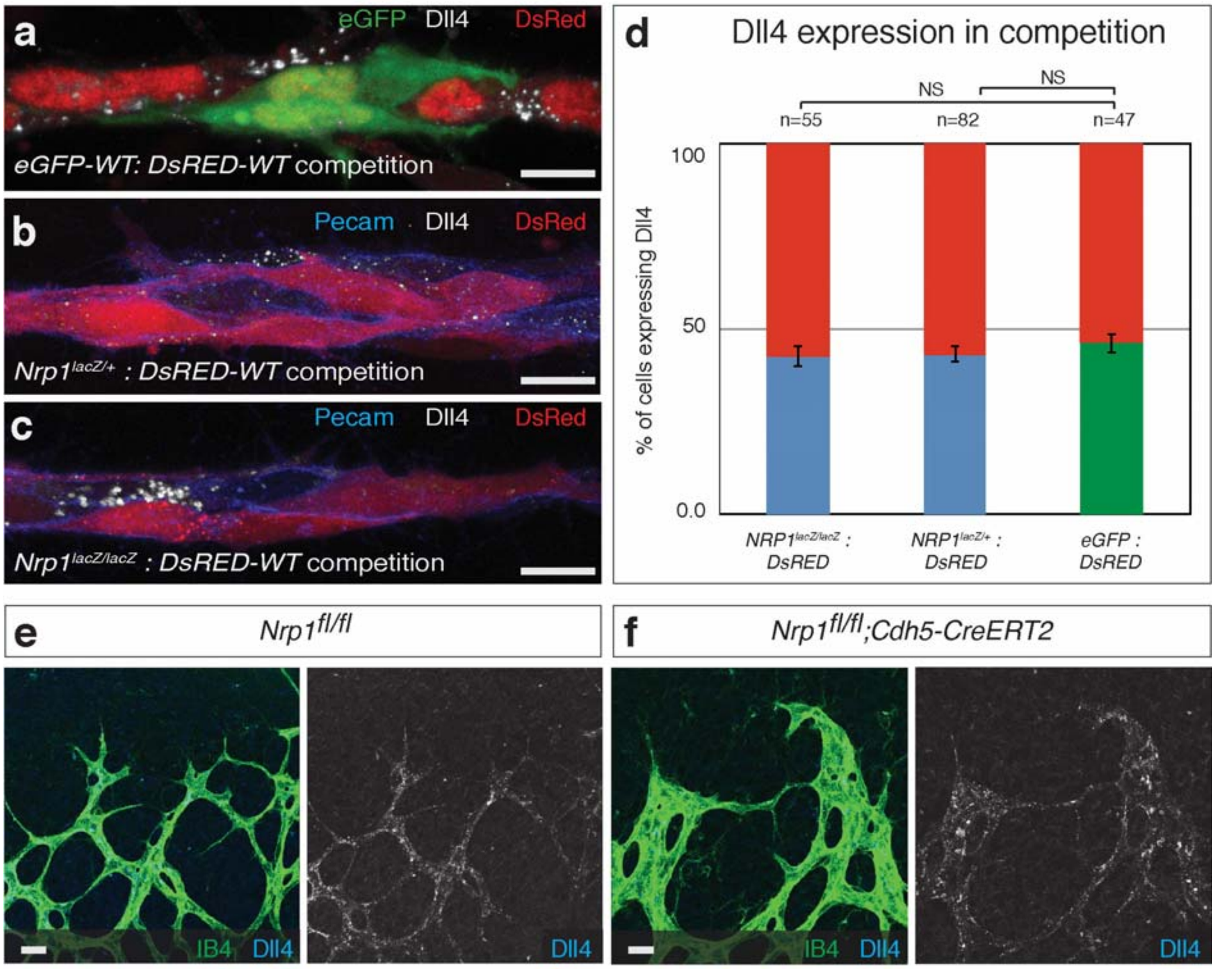

Supplementary Figure 3: Differential Nrp1 levels affect tip cell competition independently of Dll4

(a-c) Representative fluorescence microscopy images of the stalk region of a mosaic vascular sprout. (a) *DsRED-wt* endothelial cells (red) in competition with *eGFP-wt*

endothelial cells (green) showing higher levels of Dll4 (white) in the *DsRED-wt* cells, compared to the adjacent *eGFP-wt* cells. (b) *DsRED-wt* cells (red) in a competition with *Nrp1*$^{LacZ/+}$ endothelial cells (blue) showing higher levels of Dll4 (white) in the *Nrp1*$^{LacZ/+}$ cells, compared to the adjacent *DsRED-wt* cells. (c) *DsRED-wt* cells (red) in a competition with *Nrp1*$^{LacZ/LacZ}$ endothelial cells (blue) showing higher levels of Dll4 (white) in the *Nrp1*$^{LacZ/LacZ}$ cells, compared to the adjacent *DsRED-wt* cells. Scale bar: 13μm. (d) Pairwise comparison of Dll4 intensity of staining in the indicated chimeric EBs. The number of *Nrp1*$^{LacZ/LacZ}$ (n=55) or *Nrp1*$^{LacZ/+}$ (n=82) cells expressing Dll4 in a pairwise comparison with *DsRED-wt* cells is not significantly reduced to the percentage of *eGFP-wt* (n=47). n = number of sprouts counted. Values represent mean ± s.e.m. Statistical significance was assessed using a student's unpaired t-test.

(e, f) Dll4 staining in *Nrp1*$^{fl/fl}$ or *Nrp1*$^{fl/fl}$*; Cdh5-CreERT2* P5 mice, injected with 100μg of tamoxifen at P1 and P2 to induce complete deletion of *Nrp1*. Despite abnormal tips, *Nrp1*$^{fl/fl}$*; Cdh5-CreERT2* mice displayed similar Dll4 expression compared to their wt counterparts at the vascular front. Scale bar: 20μm.

Supplementary Figure 4

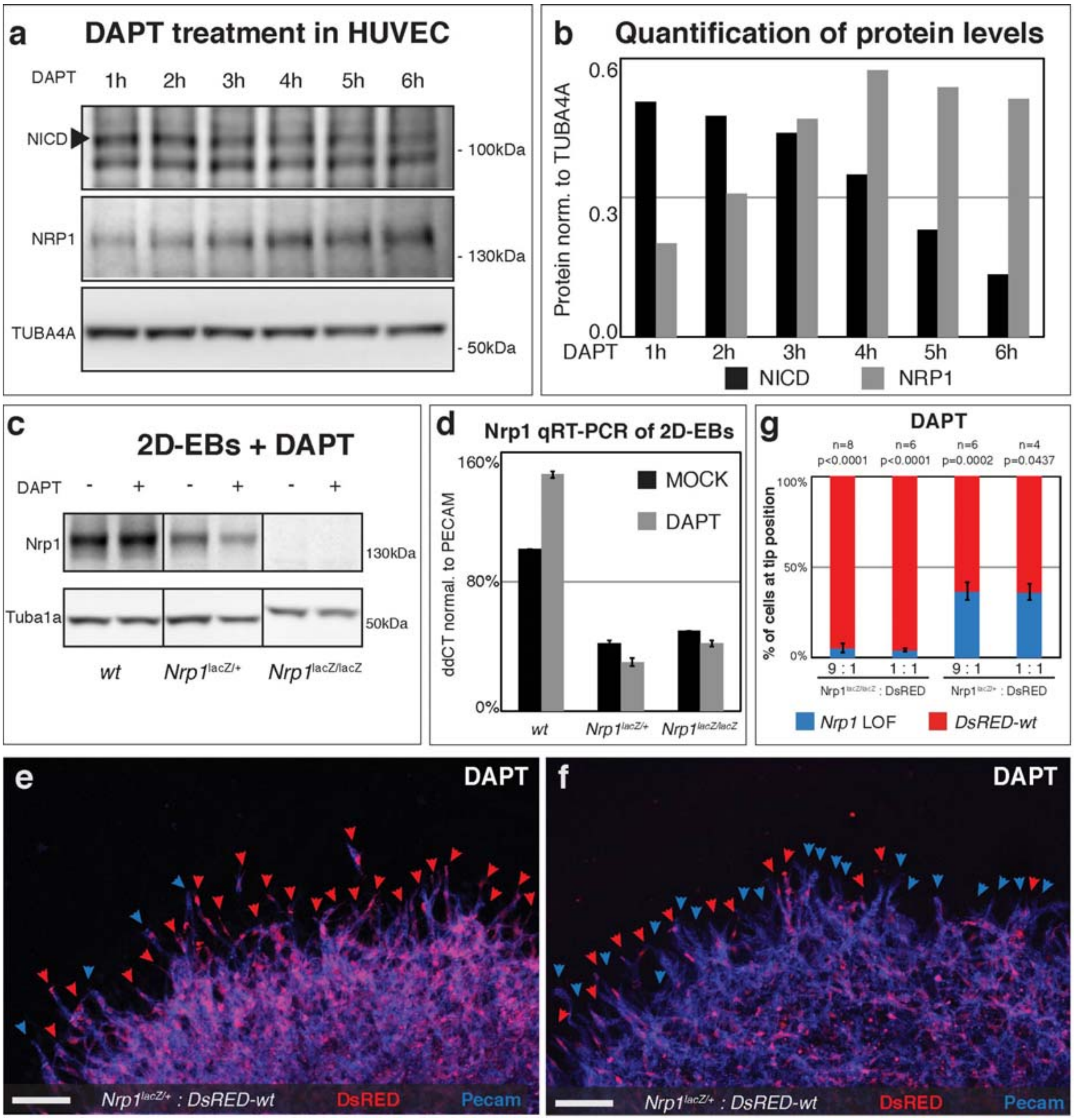

Supplementary Figure 4: Nrp1 is regulated by Notch

(a) Western-blot analysis of P4 HUVEC treated with 5µM DAPT in a time-course and stimulated with 30ng/ml VegfA$_{165}$ for 1h, a representative blot of two is shown. Full western-blots are shown in Supplementary Figure 17. (b) Quantification of NICD and NRP1 normalized to tubulin. (c) Western-blot analysis for NRP1 and tubulin of d8 2D EBs treated with 5µM DAPT starting at d5. Full western-blots are shown in Supplementary Figure 18. (d) Real-time quantitative PCR for NRP1 of d8 2D EBs treated with 5µM DAPT starting at d5, normalized to Pecam and wt MOCK control.

(e, f) Representative confocal images of chimeric EBs from wt cells and *Nrp1* deficient cells treated with 5µM DAPT. *Nrp1* deficient tip cells are indicated by blue

arrowheads, wt derived tip cells by red arrowheads. (E) *Nrp1*$^{lacZ/lacZ}$ cells (F) *Nrp1*$^{lacZ/+}$ cells mixed in a 9:1 ratio with wt cells. Scale bar: 160µm. (g) Quantification of tip cells with the indicated genotype and input levels; n = EBs per condition. n = 8 (421 tips counted) for *DsRED:Nrp1*$^{LacZ/LacZ}$ = 1:9, p<0.0001; n = 6 (1377 tips counted) for *DsRED:Nrp1*$^{LacZ/LacZ}$ = 1:1, p<0.0001; n = 6 (400 tips counted) for *DsRED:Nrp1*$^{LacZ/+}$ = 1:9, p=0.0002; n = 4 (862 tips counted) for *DsRED:Nrp1*$^{LacZ/+}$ = *1:1*□, p=0.0437. P-values were calculated using Student's unpaired t-test by comparing quantified contribution to initial percentage of input levels. Values represent mean ± s.e.m.

Supplementary Figure 5

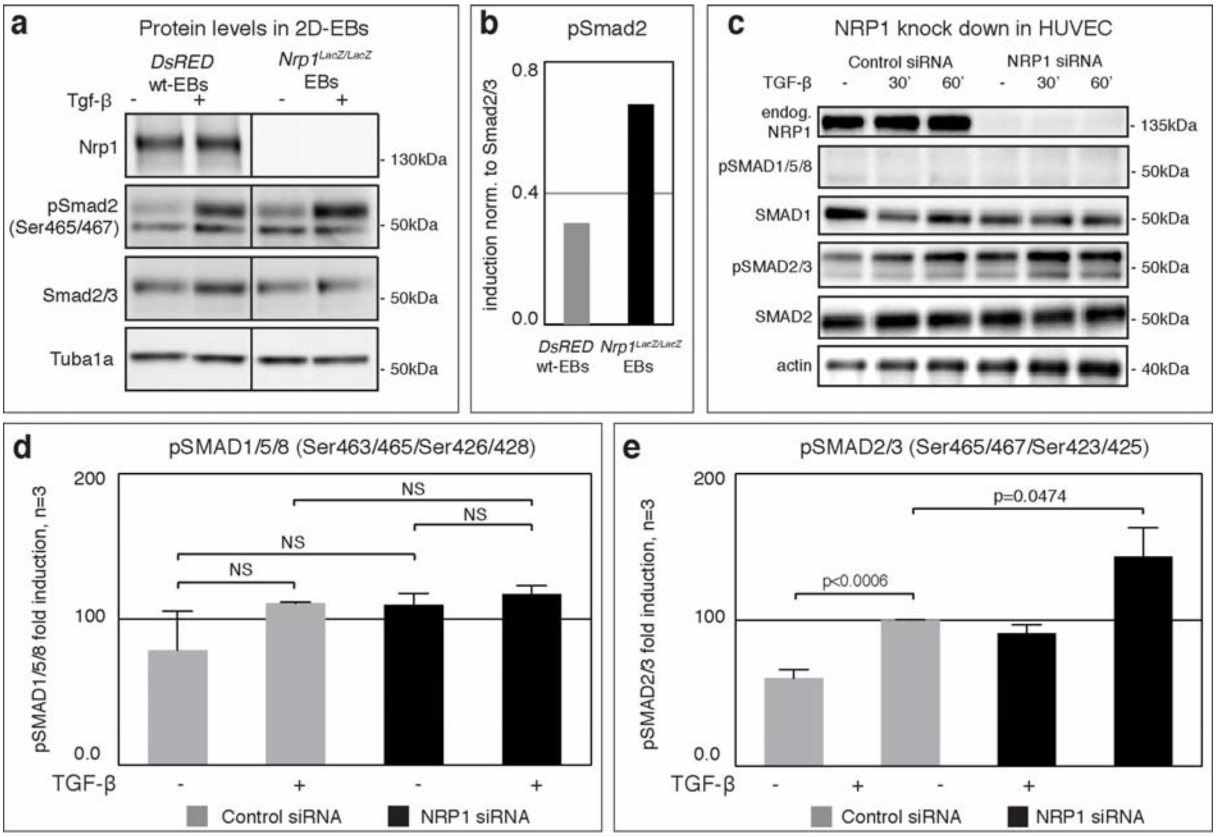

Supplementary Figure 5: Nrp1 influences Smad2/3 activation

(a, b) Western-blot analysis of d8 2D EBs derived from wt cells and *Nrp1*$^{lacZ/lacZ}$ cells, stimulated with 2ng/ml Tgf-β for 1h. Full western-blots are shown in Supplementary Figure 19. (b) Quantification of pSmad2 normalized to Smad2. (c-e) Western-blot analysis of P4 HUVEC transfected with control and NRP1 siRNA, stimulated with 2ng/ml TGF-β for 30 and 60 minutes. A representative blot of three is shown. Full western-blots are shown in Supplementary Figure 20. (d) Quantification of pSMAD1/5/8 protein normalized to SMAD1. (e) Quantification of pSMAD2/3 protein normalized to SMAD2. P values are indicated in figure.

Supplementary Figure 6

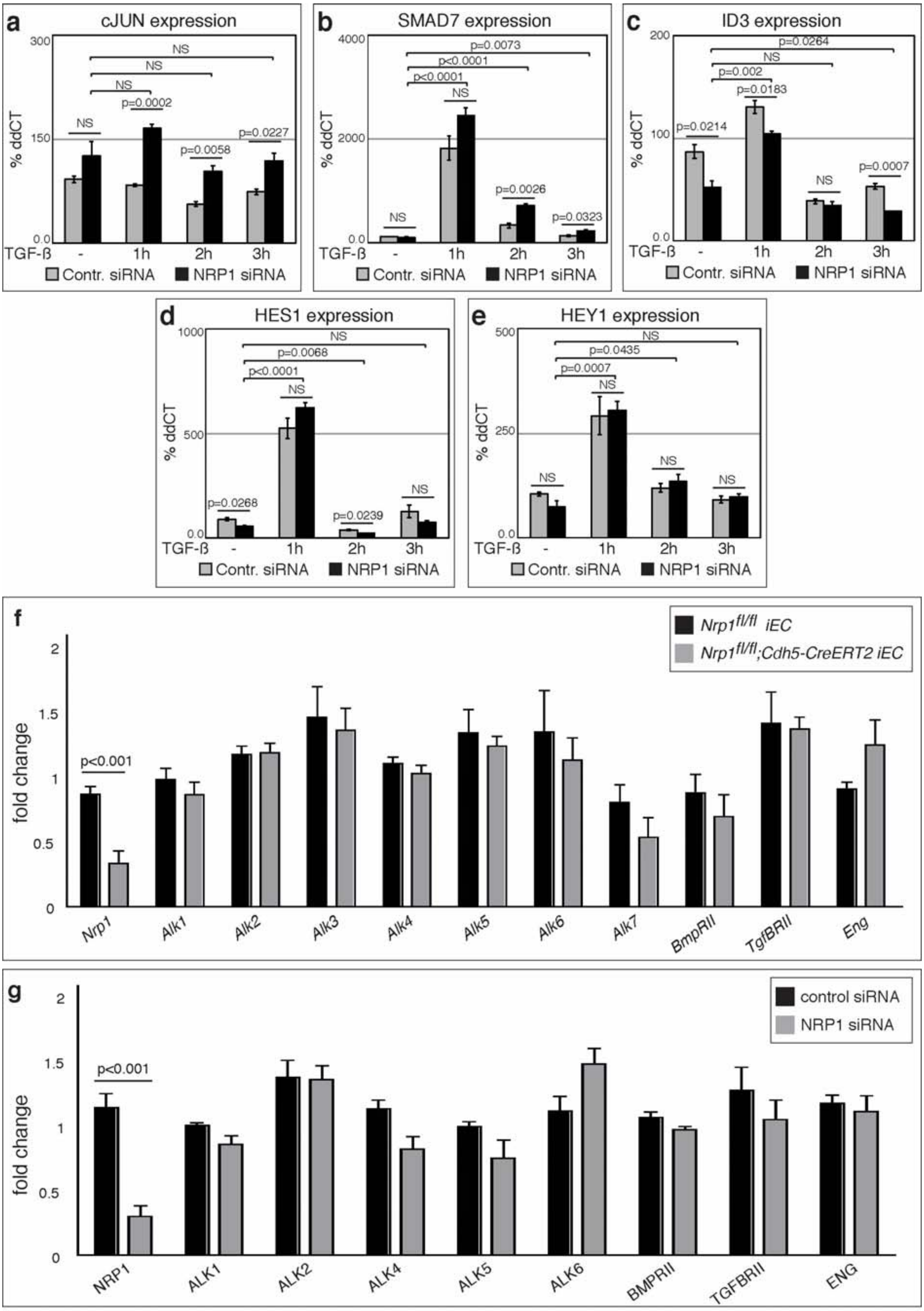

Supplementary Figure 6: Nrp1 influences Smad2/3 activation independently of Tgf-β or BMP9 receptor expression levels

(a-e) Real-time quantitative PCR for cJUN (a), SMAD7 (b), ID3 (c), HES1 (d) and HEY1 (e) of P4 HUVEC transfected with control siRNA and NRP1 siRNA for 48h. 2ng/ml TGF-β stimulation was performed in a time-course, 1h, 2h and 3h prior to cell lysis. All values are normalized to GAPDH and uninduced control siRNA. Samples have been performed as biological triplicates as well as technical triplicates. P values were calculated using Student's unpaired t-test and are indicated in the figure.

(f) Analysis of Tgf-β or BMP9 receptor expression levels in primary endothelial cells isolated from the lungs of *Nrp1*$^{fl/fl}$ or *Nrp1*$^{fl/fl}$*;Cdh5-CreERT2* mice. (g) Analysis of Tgf-β or BMP9 receptor expression levels in HUVEC transfected with control siRNA and NRP1 siRNA for 48h. All values are normalized to actin. (f, g) Samples have been performed as biological triplicates as well as technical triplicates. p<0.001. All values represent mean ± s.e.m. Statistical significance was assessed using a student's unpaired t-test.

Supplementary Figure 7

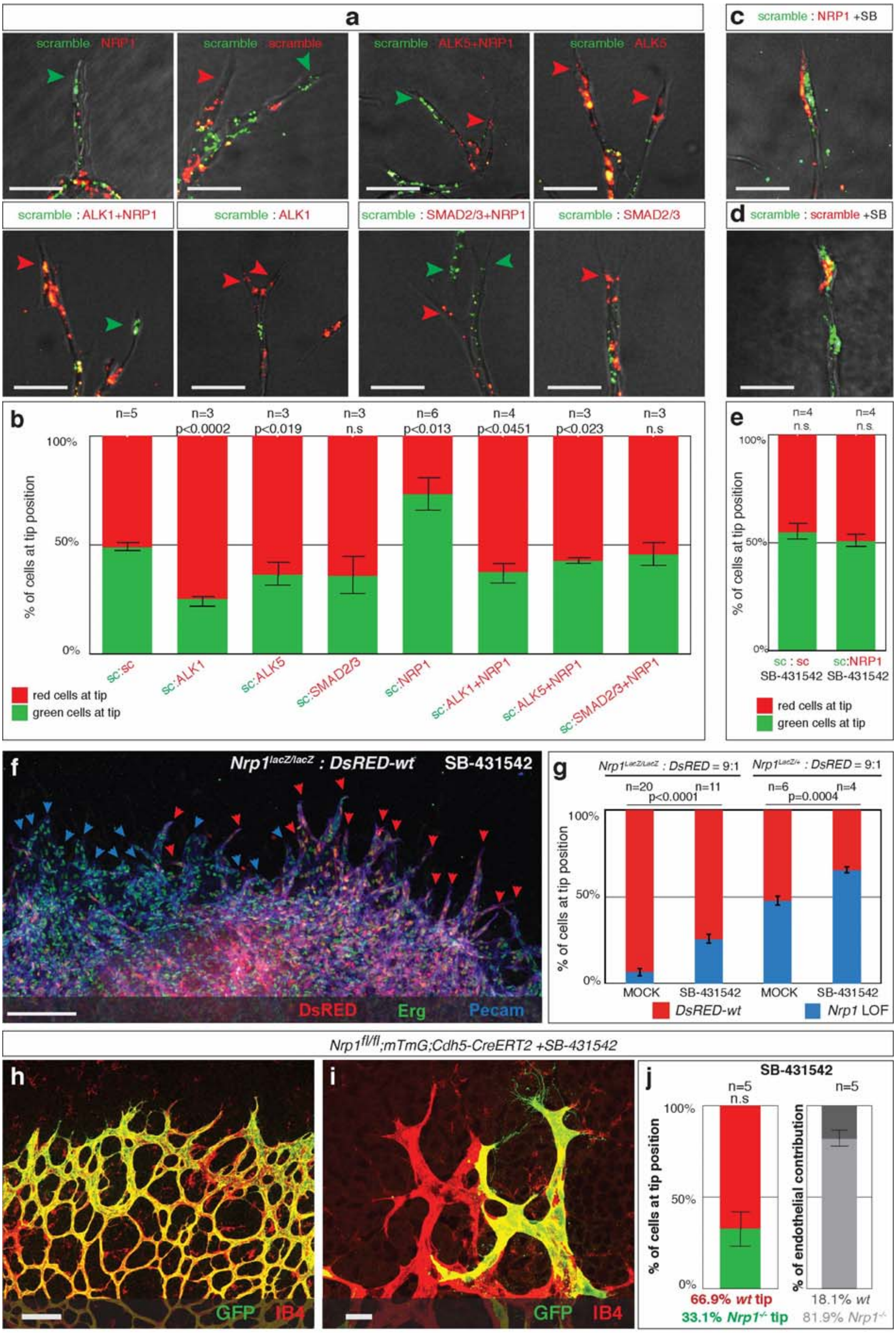

Supplementary Figure 7: Inhibition of Alk1, Alk5 or Smad2/3 rescues tip cell contribution of Nrp1 deficient cells

(a) Representative images of a mosaic sprouting assay of NRP1, ALK1, ALK5, SMAD2/3 siRNA-transfected HUVECs (red) in competition with scrambled siRNA-transfected HUVECs (green); as well as NRP1/ALK1, NRP1/ALK5, NRP1/SMAD2/3 siRNA-transfected HUVECs (red) in competition with scrambled siRNA-transfected HUVECs (green). Arrowheads indicate tip cell colour. (c, d) Representative images of a mosaic sprouting assay of scramble siRNA (d) or NRP1 siRNA (c) transfected HUVEC in competition with scramble siRNA-transfected HUVECs (green) after treatment with 10µM SB-431542. (a, c, d) Scale bar: 20µm. (b, e) Quantification of tip cell contribution, n = number of individual experiments as indicated in figure. Statistical significance was assessed by comparing the percentage of green cells at the tip in sc:sc to the percentage of green cells at the tip in each experiment. p values indicated in figure.

(f) Representative confocal image of chimeric EBs composed of 1 part wt (tip cells indicated by blue arrowheads) and 9 parts *Nrp1*$^{lacZ/lacZ}$ cells (tip cells indicated by blue arrowheads), immunolabeled for Pecam and Erg, treated with 10µM SB-431542 once a day starting at day 6. Scale bar: 160µm. (g) Quantification of tip cells with the indicated genotype; n = EBs per condition; n = 20 (613 tips counted in total) for *DsRED:Nrp1*$^{LacZ/LacZ}$ = 1:9, MOCK; n = 11 (223 tips counted) for *DsRED:Nrp1*$^{LacZ/LacZ}$ = 1:9, SB-431542; p<0.0001; n = 6 (504 tips counted) for *DsRED:Nrp1*$^{LacZ/+}$ = 1:9, MOCK; n = 4 (324 tips counted) for *DsRED:Nrp1*$^{LacZ/+}$ = 1:19, SB-431542; p=0.0004). P-values were calculated using Student's unpaired t-test by comparing quantified contribution to initial percentage of input levels. Values represent mean ± s.e.m.

(h, i) Retinas of *Nrp1*$^{fl/fl}$*; mTmG; Cdh5-CreERT2* mice injected with 30µg tamoxifen at P1, treated with 20mg/kg SB-431542 at P3 and P4, retinas were assayed P5. Unrecombined wt cells labeled with Isolectin-B4 only, recombined *Nrp1* deficient cells express GFP. (h) Representative overview of the sprouting front, scale bar: 100µm, (i) higher magnification, scale bar: 20µm. (j) Quantification of recombined *Nrp1* deficient cells at the tip, normalized to overall contribution of cells to the endothelium. N=5 (number of retinas analyzed). Statistical significance was determined by comparing the proportion of Nrp1 deficient (green) cells at the tip to the total proportion of Nrp1 deficient (green) cells. p = n.s. (b, e, g, j) Values represent mean ± s.e.m. Statistical significance was assessed using a student's unpaired t-test.

Supplementary Figure 8

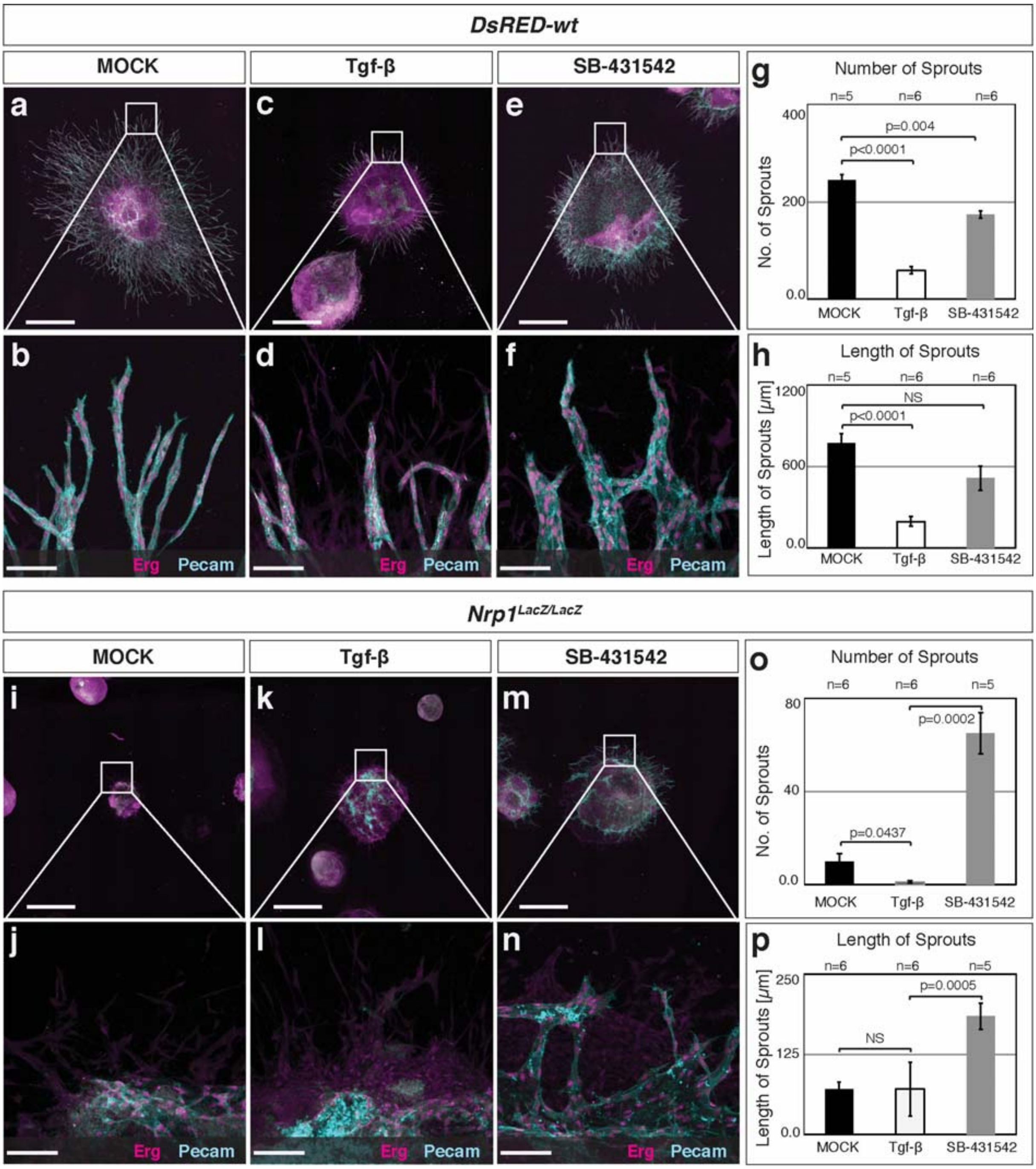

Supplementary Figure 8: Tgf-β inhibits sprouting and Alk5 inhibition rescues sprouting in Nrp1 deficient EBs

(a-f) Representative confocal images of EBs derived wt ES cells. Sprouting vasculature is highlighted with Pecam and Erg. Treatment started at d6 continuously until harvest at d10. (a) Untreated EBs, magnification in (b). (c) Treatment with 2ng/ml Tgf-β, magnification in (d). (e) Treatment with 10μM SB-431542, magnification in (f). Scale bar: 840μm (a, c, e), 84μm (b, d, f). (g) Quantification of sprouts. Values represent means ± s.e.m.; n=5, untreated; n=6, Tgf-β; n=6,

SB431542; (n = number of EBs used for quantification). (h) Length of sprouts in μm. Values represent means ± s.e.m.; n=5, untreated; n=6, Tgf-β; n=6, SB431542 (n = number of EBs). p values indicated in figure. (i-p) Representative confocal images of the sprouting vasculature of EBs derived from *Nrp1*$^{lacZ/lacZ}$ ES cells stained for Pecam and Erg. Treatment started at d6 until harvest at d11. (i) Untreated EBs, magnification in (j). (k) Treatment with 2ng/ml Tgf-β, magnification in (l). (m) Treatment with 10μM SB-431542, magnification in (n). Scale bar: 840μm (I, K, M), 84μm (J, L, N). (o) Quantification of sprouts. Values represent mean ± s.e.m.; n=5, untreated; n=5, Tgf-β; n=6, SB-431542; (n = number of EBs used for quantification). (p) Length of sprouts in μm. Values represent means ± s.e.m.; n=5, untreated; n=5, Tgf-β; n=6, SB-431542 (n = number of EBs). p values indicated in figure. Statistical significance was assessed using a student's unpaired t-test.

Supplementary Figure 9

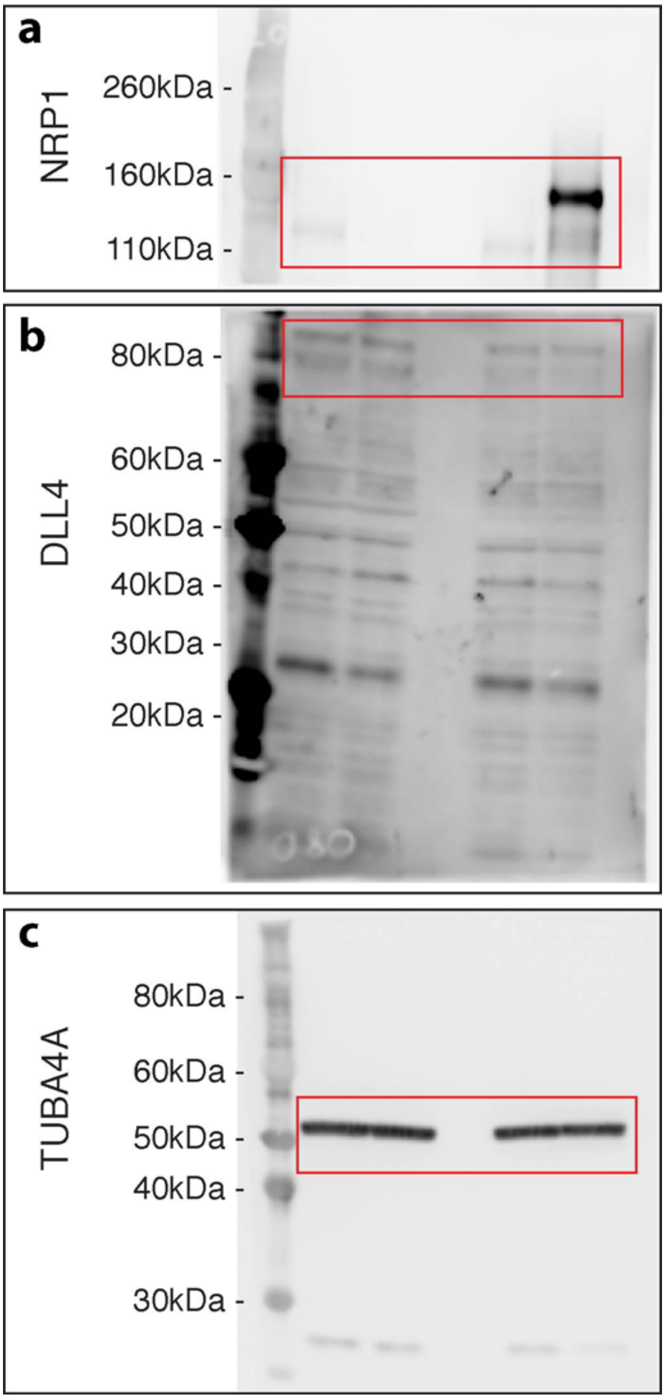

Supplementary Figure 9: Western-blots used in Figure 2b

(a-c) P4 HUVEC cells transfected with control siRNA, NRP1 siRNA, control-GFP-construct, NRP1-GFP-His-construct. NRP1 and DLL4 protein levels were assessed 24h after transfection by western-blot. Western-blot for (a) NRP1, (b) DLL4 and (c) TUA4A. Panels used in the main figure are marked with a red square.

Supplementary Figure 10

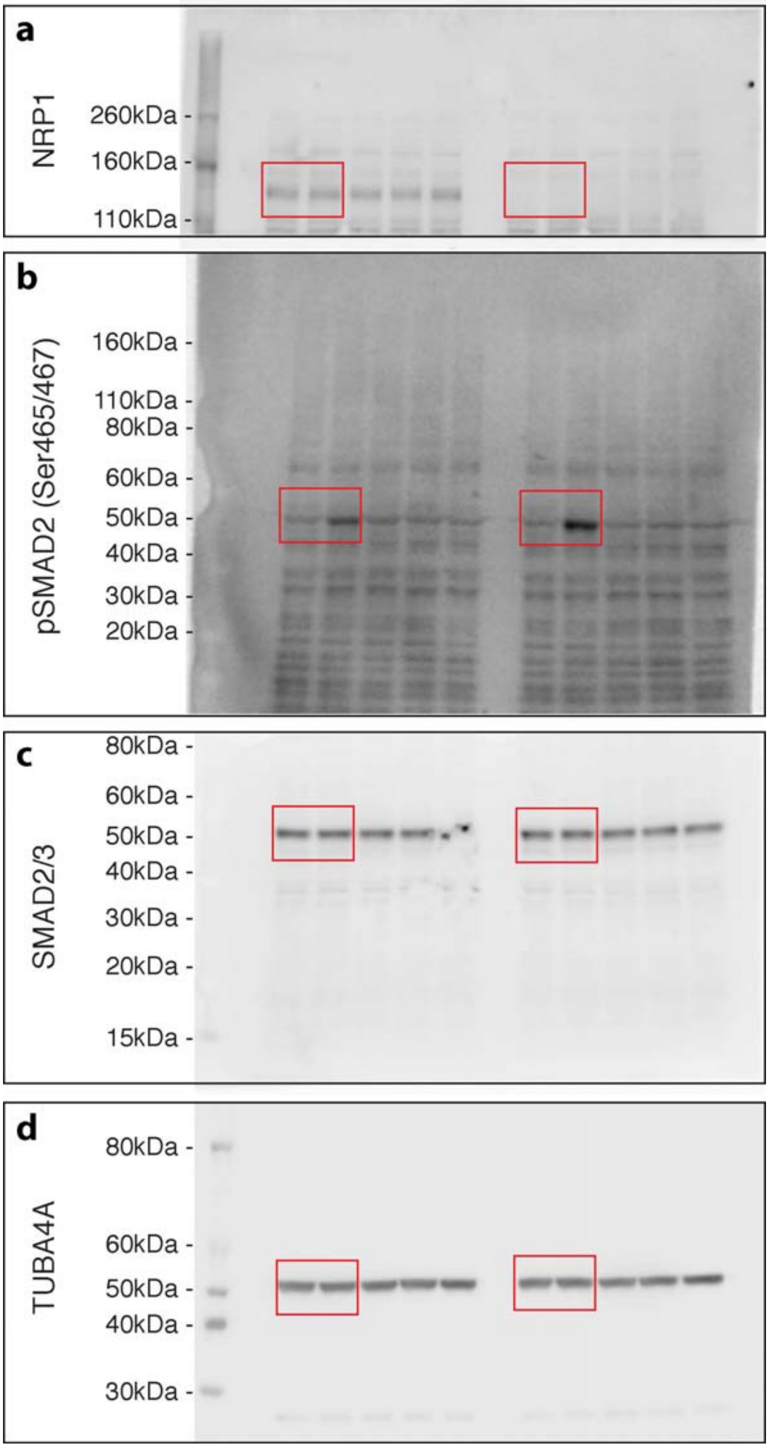

Supplementary Figure 10: Western-blots used in Figure 5d

(a-d) P4 HUVEC transfected with control siRNA and NRP1 siRNA, with or without stimulation with 2ng/ml TGF-β for 1h. Western-blot for (a) NRP1, (b) pSMAD2 (Ser465/467), (c) SMAD2/3 and (d) TUBA4A. Panels used in the main figure are marked with a red square.

Supplementary Figure 11

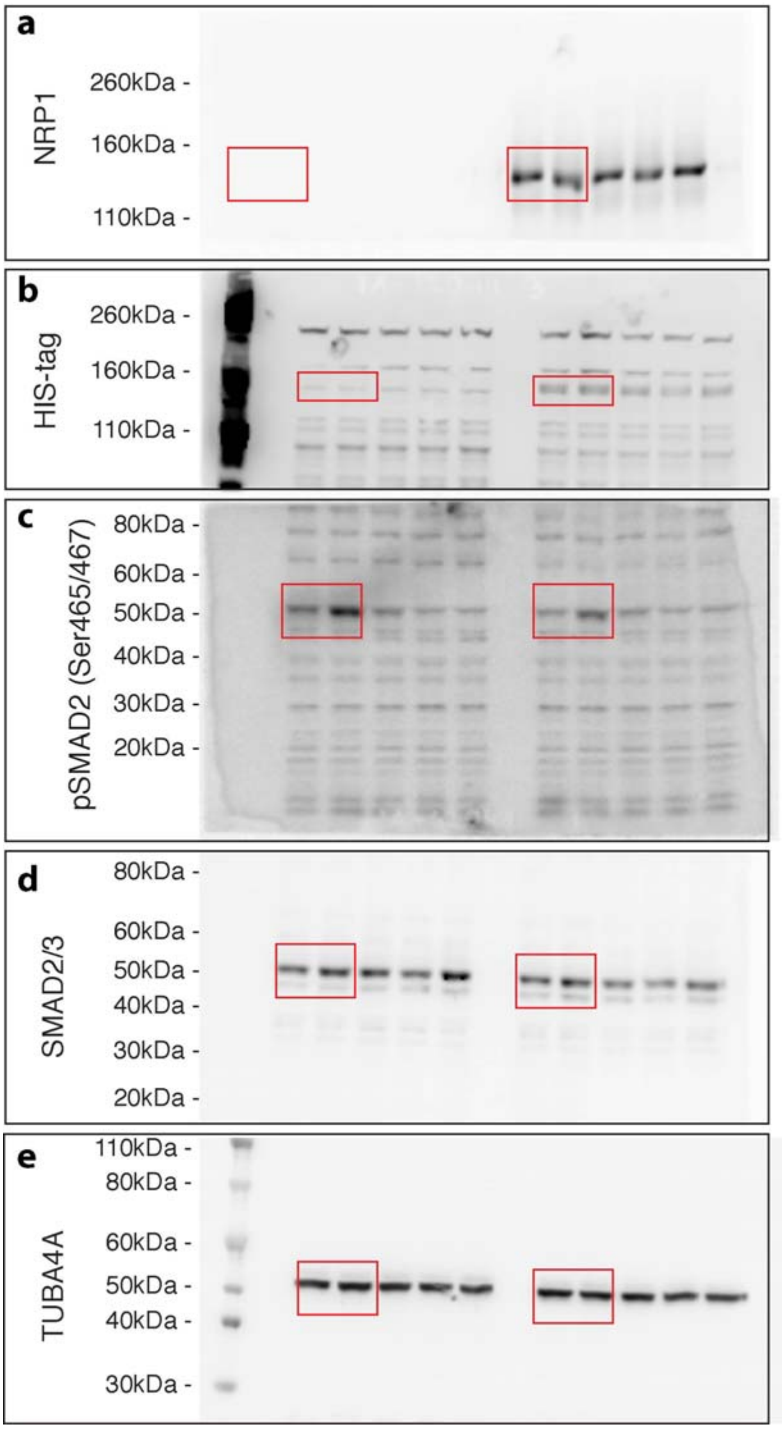

Supplementary Figure 11: Western-blots used in Figure 5f

(a-e) P4 HUVEC transfected with control-GFP- and NRP1-GFP-His construct for 24h, with or without stimulation with 2ng/ml TGF-β for 1h. Western-blot for (a) NRP1, (b) His-tag, (c) pSMAD2 (Ser465/467), (d) SMAD2/3 and (e) TUBA4A. Panels used in the main figure are marked with a red square.

Supplementary Figure 12

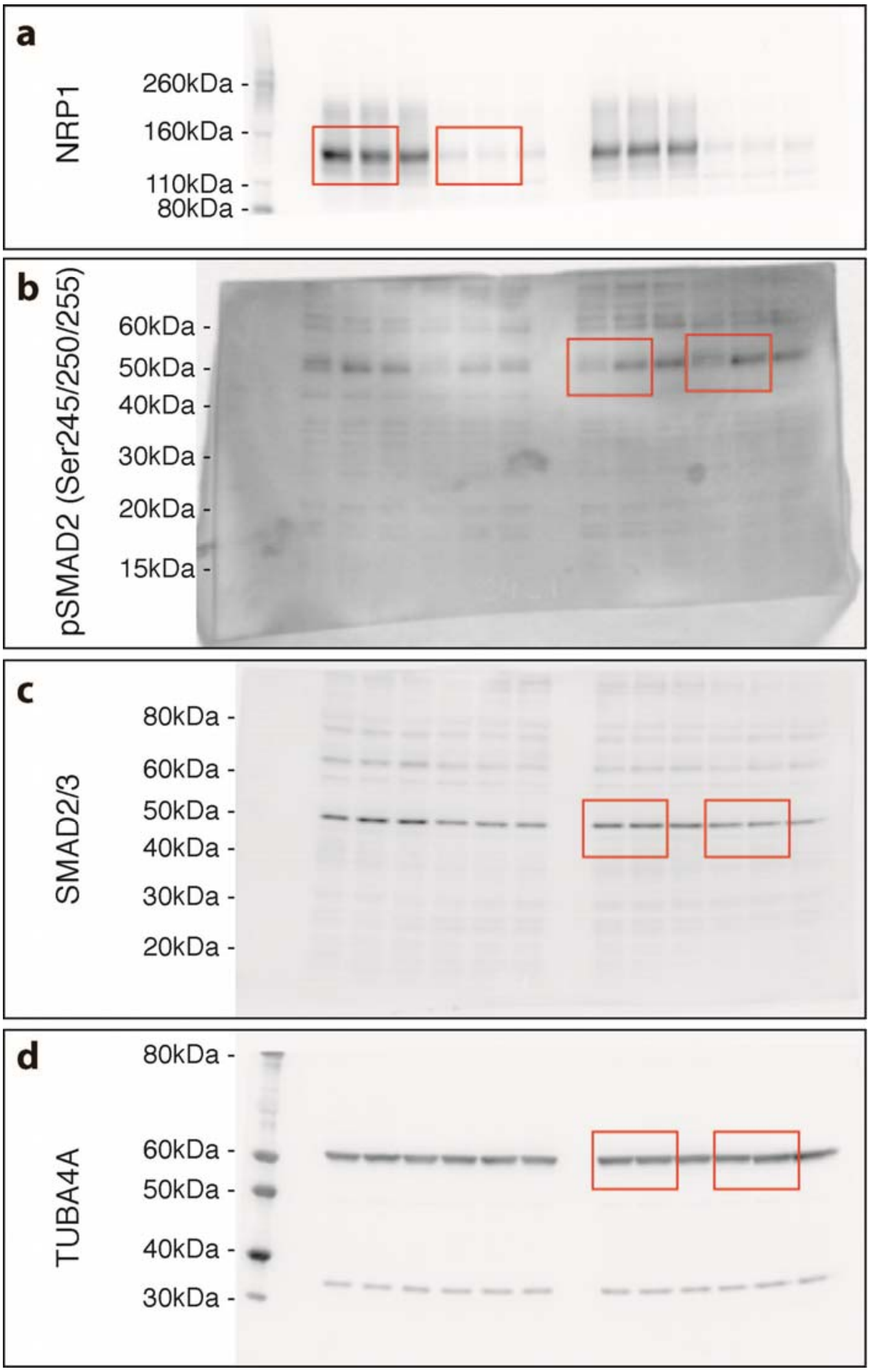

Supplementary Figure 12: Western-blots used in Figure 5h

(a-d) P4 HUVEC transfected with control siRNA and NRP1 siRNA, with or without stimulation with 2ng/ml TGF-β for 1h. Western-blot for (a) NRP1, (b) pSMAD2 (Ser245/250/255), (c) SMAD2/3 and (d) TUBA4A. Panels used in the main figure are marked with a red square.

Supplementary Figure 13

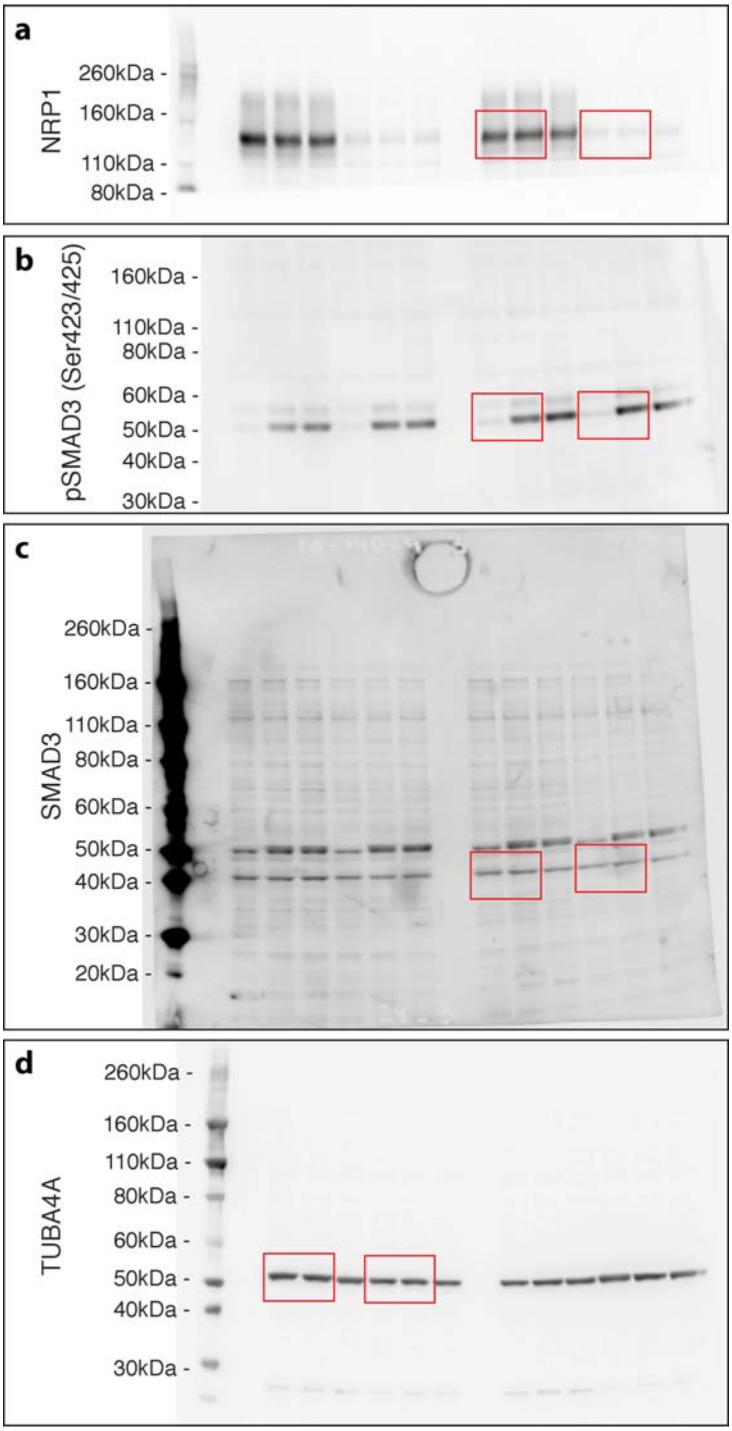

Supplementary Figure 13: Western-blots used in Figure 5j

(a-d) Western-blot analysis of proteins from P4 HUVEC transfected with control siRNA and NRP1 siRNA, with or without stimulation with 2ng/ml TGF-[illegible] for 1h. Western-blot for (a) NRP1, (b) pSMAD3 (Ser423/425), (c) SMAD3 and (d) TUBA4A. Panels used in the main figure are marked with a red square.

Supplementary Figure 14

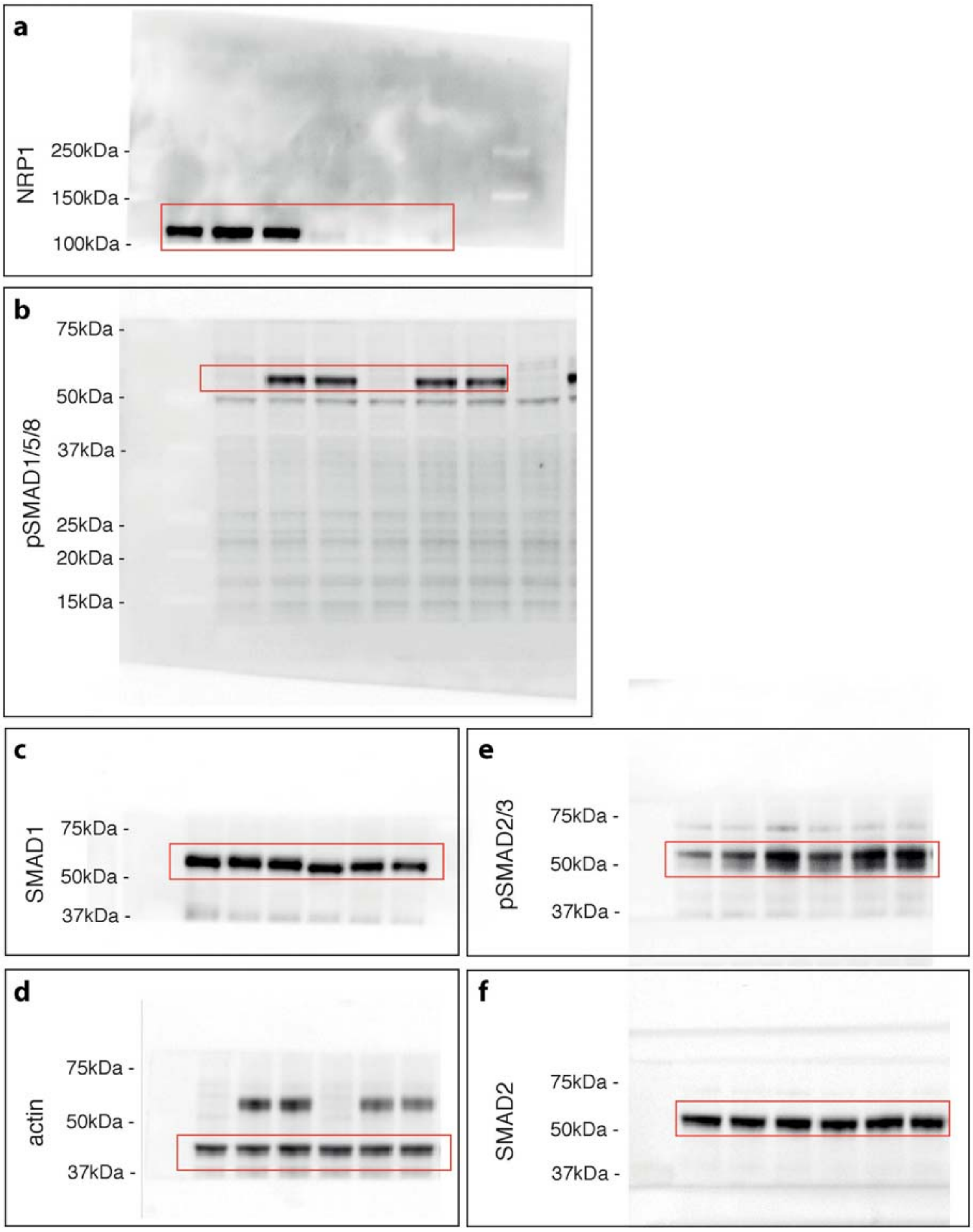

Supplementary Figure 14: Western-blots used in Figure 5l

(a-f) P4 HUVEC transfected with control siRNA and NRP1 siRNA, with or without stimulation with 10ng/ml BMP-9 for 15 and 30 minutes. Western-blot for (a) NRP1, (b) pSMAD1/5/8, (c) SMAD1, (d) ACTIN, (e) pSMAD2/3 and (f) SMAD2. Panels used in the main figure are marked with a red square.

Supplementary Figure 15

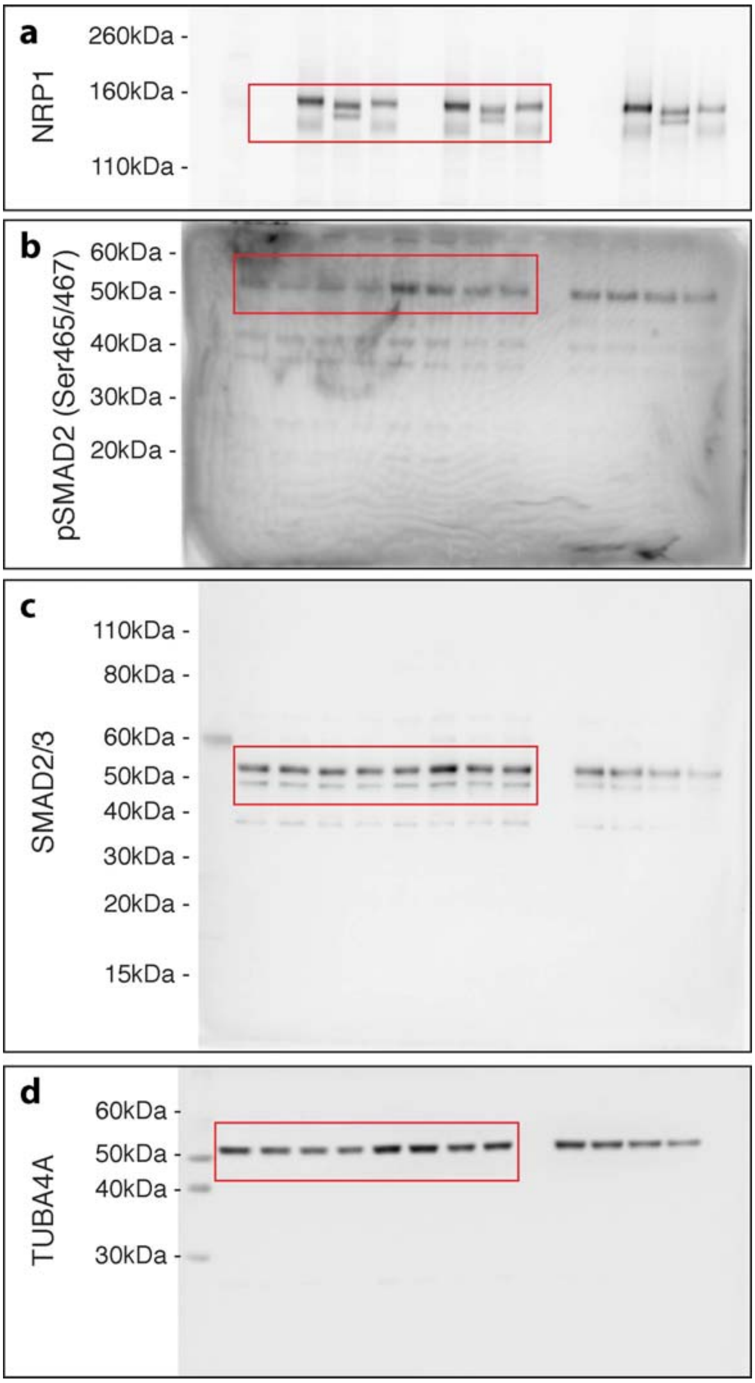

Supplementary Figure 15: Western-blots used in Figure 6e

(a-d) P4 HUVEC transfected with control-GFP-construct, full length NRP1-GFP-His-construct, cytoplasmic domain deleted NRP1-dCY-GFP-His-construct and SEA domain deleted NRP1-dSEA-GFP-His-construct for 24h prior stimulation with 2ng/ml TGF-β for 1h. Western-blot for (a) NRP1, (b) pSMAD2 (Ser465/467), (c) SMAD2/3 and (d) TUBA4A. Panels used in the main figure are marked with a red square.

Supplementary Figure 16

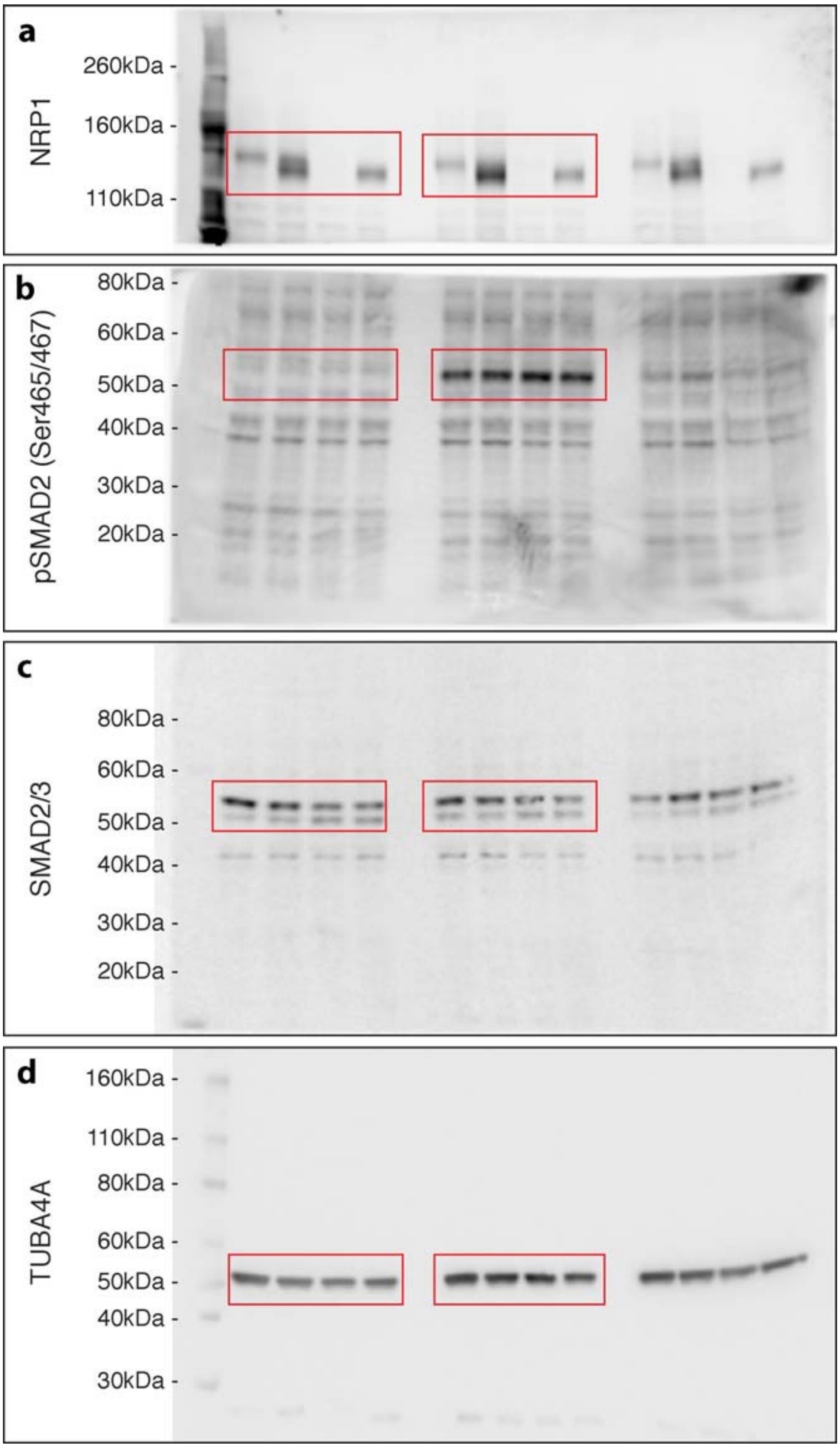

Supplementary Figure 16: Western-blots used in Figure 6g

(a-d) P4 HUVEC transfected with control siRNA and NRP1 siRNA for 48h treated with 10nM recombinant NRP1 1h prior stimulation with 2ng/ml TGF-β for 1h. Western-blot for (a) NRP1, (b) pSMAD2 (Ser465/467), (c) SMAD2/3 and (d) TUBA4A. Panels used in the main figure are marked with a red square.

Supplementary Figure 17

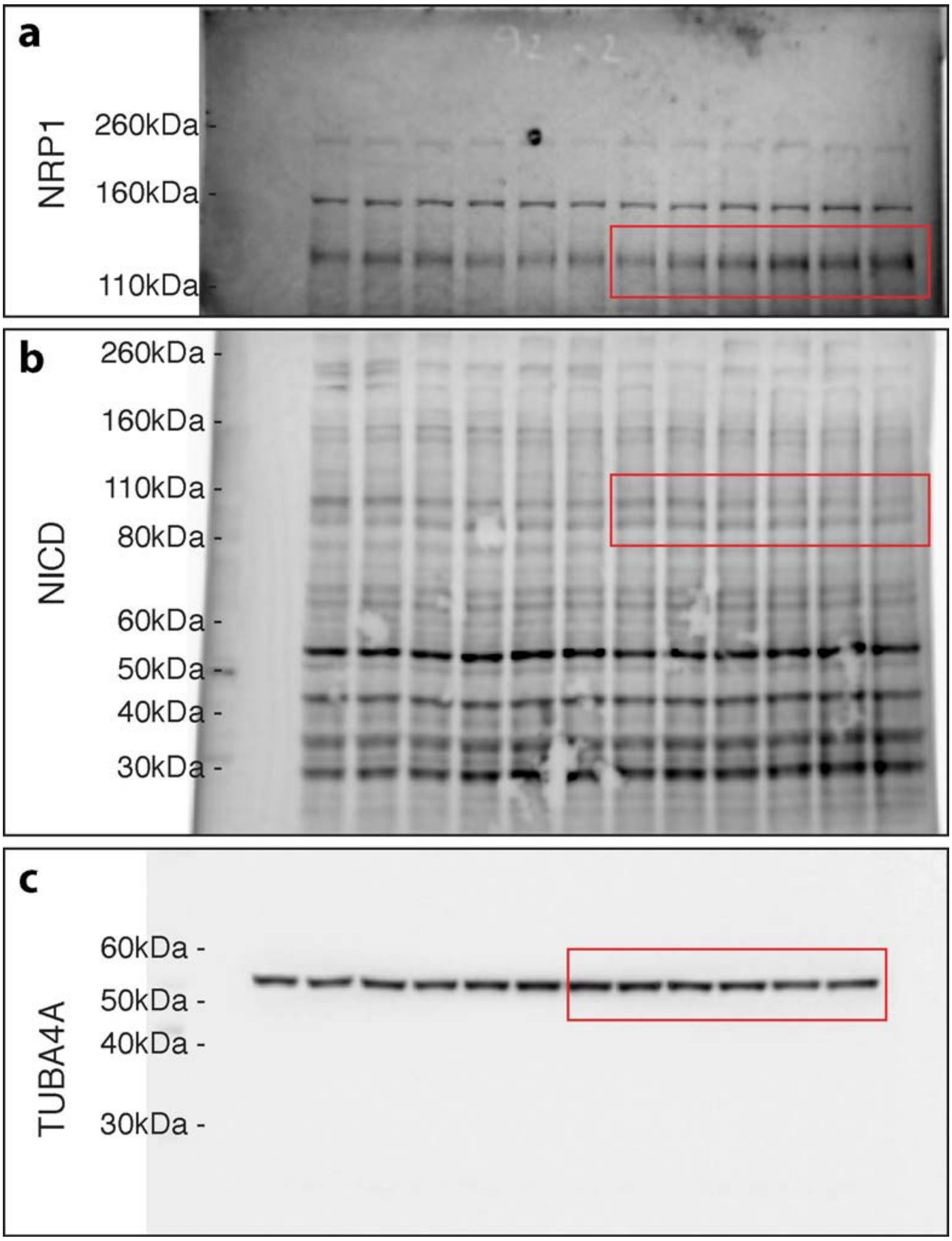

Supplementary Figure 17: Western-blots used in Supplementary Figure 4a

(a-c) P4 HUVEC treated with 5µM DAPT in a time-course and stimulated with 30ng/ml $VegfA_{165}$ for 1h. Western-blot for (a) NRP1, (b) NICD and (c) TUBA4A. Panels used in the main figure are marked with a red square.

Supplementary Figure 18

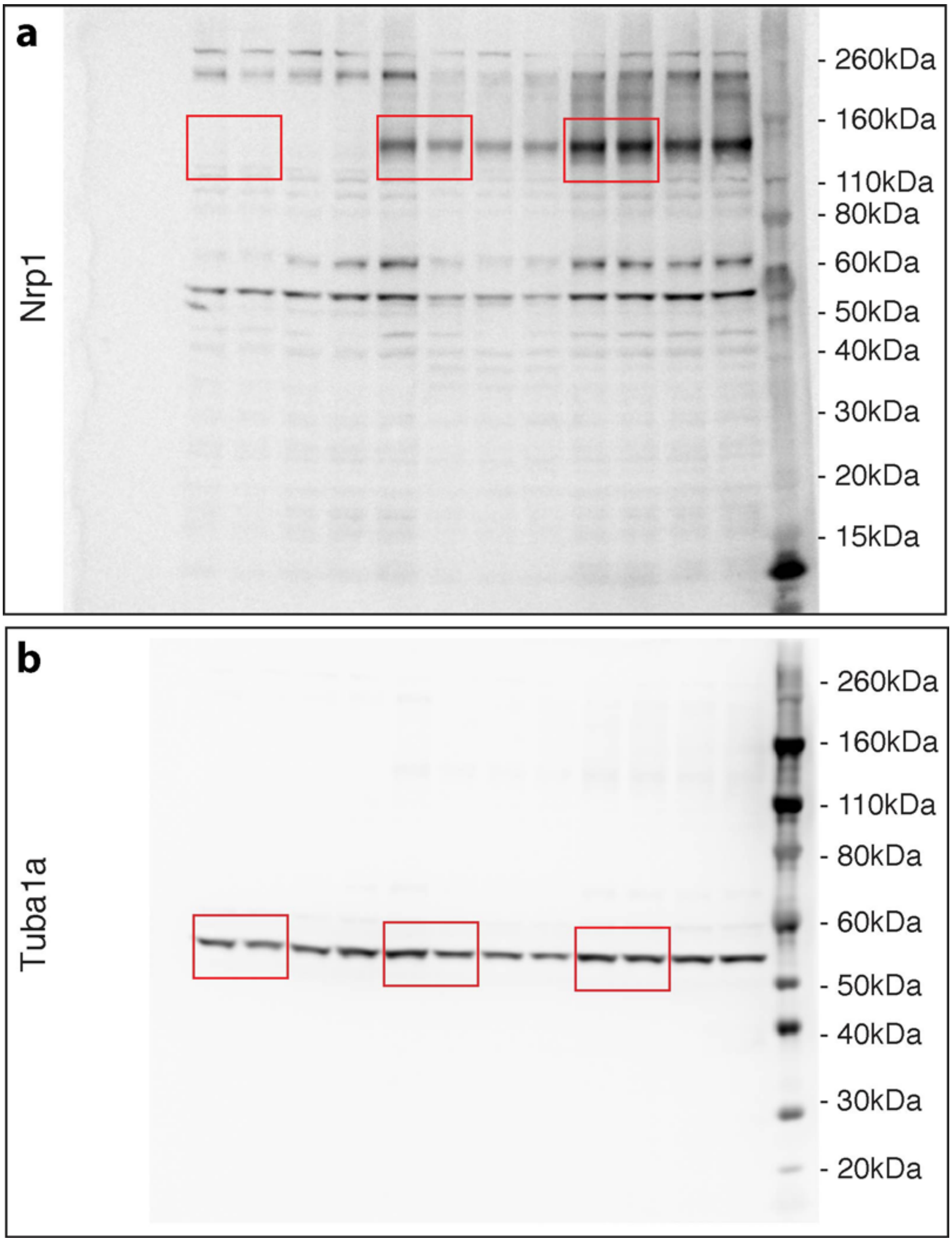

Supplementary Figure 18: Western-blots used in Supplementary Figure 4c

(a-b) D8 2D EBs treated with 5μM DAPT starting at d5. Western-blot for (a) Nrp1 and (b) Tuba1a. Panels used in the main figure are marked with a red square.

Supplementary Figure 19

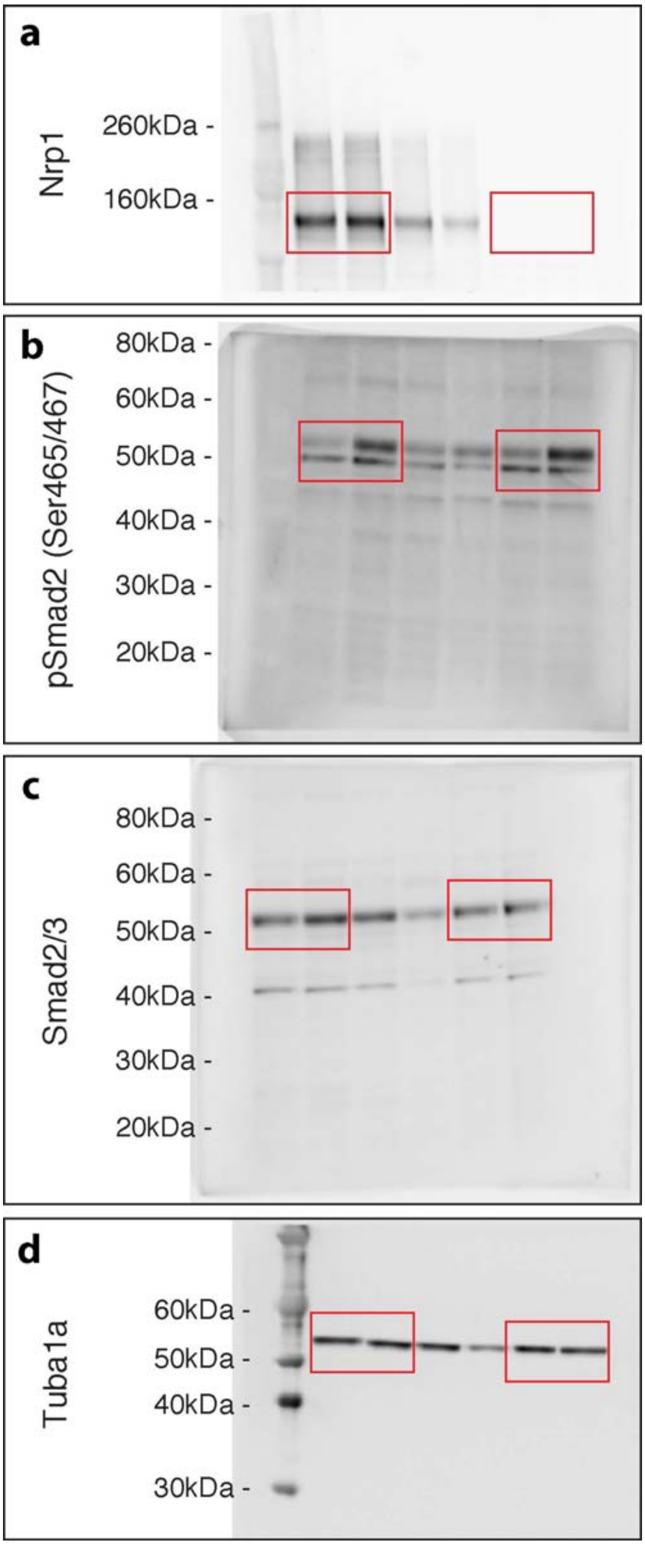

Supplementary Figure 19: Western-blots used in Supplementary Figure 5a

(a-d) D8 2D EBs derived from wt cells and *Nrp1*$^{lacZ/lacZ}$ cells, stimulated with 2ng/ml Tgf-β for 1h. Western-blot for (a) Nrp1, (b) pSmad2 (Ser465/467), (c) Smad2/3 and (d) Tuba1a. Panels used in the main figure are marked with a red square.

Supplementary Figure 20

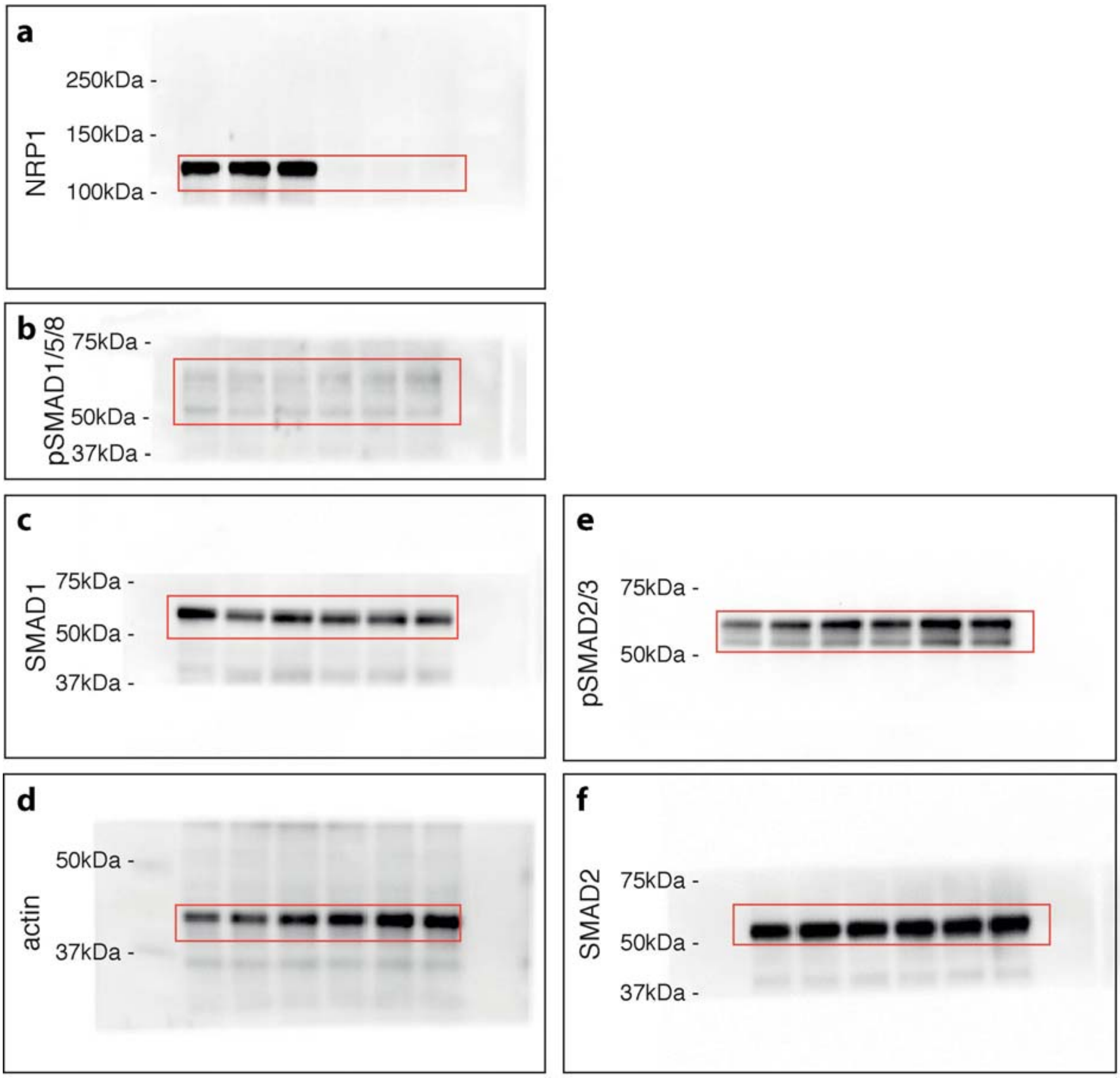

Supplementary Figure 20: Western-blots used in Supplementary Figure 5c

(a-f) P4 HUVEC transfected with control and NRP1 siRNA, stimulated with 2ng/ml TGF-β for 30 and 60 minutes. Western-blot for (a) NRP1, (b) pSMAD1/5/8, (c) SMAD1, (d) ACTIN, (e) pSMAD2/3 and (f) SMAD2. Panels used in the main figure are marked with a red square.
